# Gene expression profiles of human melanoma cells with different invasive potential reveal TSPAN8 as a novel mediator of invasion

**DOI:** 10.1038/sj.bjc.6605994

**Published:** 2010-11-16

**Authors:** O Berthier-Vergnes, M El Kharbili, A de la Fouchardière, T Pointecouteau, P Verrando, A Wierinckx, J Lachuer, F Le Naour, J Lamartine

**Affiliations:** 1 Université de Lyon, Lyon F-69003, France; 2 Université Lyon 1, Bâtiment Grégor Mendel, 43 Boulevard du 11 Novembre 1918, Lyon F-69003, France; 3 CNRS, UMR5534, Centre de Génétique Moléculaire et Cellulaire, Villeurbanne F-69622, France; 4 Centre Léon Bérard, Département d’Anatomie et Cytologie Pathologiques, Lyon F-69008, France; 5 INSERM UMR 911 – CRO2, Faculté de Médecine Timone, Université de la Méditerranée, Marseille, France; 6 Université de Lyon, Lyon 1, Faculté de Médecine Lyon-Est, Rue Guillaume Paradin, Lyon cedex 08 69372, France; 7 ProfileXpert, Bron F-69677, France; 8 Inserm, U602, Villejuif F-94807, France; 9 Université Paris-Sud, Institut André Lwoff, Villejuif F-94807, France

**Keywords:** tetraspanin 8, marker, invasion, melanoma

## Abstract

**Background:**

Metastatic melanoma requires early detection, being treatment resistant. However, the earliest events of melanoma metastasis, and especially of dermal invasion, remain ill defined.

**Results and methods:**

Gene expression profiles of two clonal subpopulations, selected from the same human melanoma cell line, but differing in ability to cross the dermal–epidermal junction in skin reconstructs, were compared by oligonucleotide microarray. Of 26 496 cDNA probes, 461 were differentially expressed (>2-fold; *P*< 0.001), only 71 genes being upregulated in invasive cells. Among them, TSPAN8, a tetraspanin not yet described in melanoma, was upregulated at mRNA and protein levels in melanoma cells from the invasive clone, as assessed by RT–PCR, flow cytometry and western blot analysis. Interestingly, TSPAN8 was the only tetraspanin in which overexpression correlated with invasive phenotype. Flow cytometry of well-defined melanoma cell lines confirmed that TSPAN8 was exclusively expressed by invasive, but not non-invasive melanoma cells or normal melanocytes. Immunohistochemistry revealed that TSPAN8 was expressed by melanoma cells in primary melanomas and metastases, but not epidermal cells in healthy skin. The functional role of TSPAN8 was demonstrated by silencing endogenous TSPAN8 with siRNA, reducing invasive outgrowth from tumour spheroids within matrigel without affecting cell proliferation or survival.

**Conclusion:**

TSPAN8 expression may enable melanoma cells to cross the cutaneous basement membrane, leading to dermal invasion and progression to metastasis. TSPAN8 could be a promising target in early detection and treatment of melanoma.

Melanoma is the leading cause of death of all skin diseases. Genetic predisposition and intensive sun exposure are risk factors for melanoma development (Miller and Mihm, 2006). Despite worldwide prevention efforts, melanoma incidence is increasing faster than any other type of cancer (Miller and Mihm, 2006). Successful treatment depends on early detection, as the metastatic form is resistant to current therapies (Miller and Mihm, 2006).

The cascade of events driving metastasis is a highly regulated multistep process, in which melanoma cells first proliferate within the epidermis (radial growth phase (RGP)), then penetrate through the dermal–epidermal junction, deeply invade the underlying dermis (vertical growth phase (VGP)), enter the circulatory system and colonise the target organ (Miller and Mihm, 2006). Although the Breslow thickness of the primary tumour remains the single most powerful prognostic factor, thin melanomas may develop metastases, whereas thick melanomas may sometimes fail to progress (Gimotty *et al*, 2004). Identifying biomarkers associated with conversion from RGP to VGP in primary lesions is therefore pivotal for early prediction of clinical outcome and development of anti-invasive therapies.

Several studies have used microarrays for genome scale analysis in clinical specimens and cell lines, especially to identify subsets of genes that contribute to melanoma genesis and progression (Bittner *et al*, 2000; Clark *et al*, 2000; Hoek *et al*, 2004; de Wit *et al*, 2005; Haqq *et al*, 2005; Smith *et al*, 2005; Talantov *et al*, 2005; Winnepenninckx *et al*, 2006; Jaeger *et al*, 2007; Ryu *et al*, 2007). Interestingly, several research groups reported that increased expression of genes involved in extracellular matrix assembly and in regulating the cytoskeleton correlated with higher metastatic capacity (Bittner *et al*, 2000; Clark *et al*, 2000; Winnepenninckx *et al*, 2006). However, many of the model systems used to study melanoma metastasis bypass the earliest events, and especially dermal invasion. Consequently, this critical first step in the metastatic process has been less well studied than later stages.

A few recent studies point to a common metastasis signature emerging as primary melanoma tumours thicken (Haqq *et al*, 2005; Winnepenninckx *et al*, 2006; Jaeger *et al*, 2007; Ryu *et al*, 2007), strengthening the concept that metastatic subpopulations of cells pre-exist within a primary tumour (Fidler, 2002). In line with this, we previously reported that subpopulations of melanoma cells, selected from a non-aggressive parental cell line for their ability to bind the peanut agglutinin lectin (PNA), generated lung metastases when injected subcutaneously into neonatal immune-suppressed rats (Doré *et al*, 1994; Zebda *et al*, 1994, 1995). More importantly, such PNA-positive cells are able to cross the dermal–epidermal junction by degrading collagen IV and VII, and thus colonise the dermis of human skin reconstructs (Béchetoille *et al*, 2000; Goidin *et al*, 2001). In sharp contrast, PNA-negative melanoma cells only proliferate in the epidermis near the basement membrane, without infiltrating the dermis (Béchetoille *et al*, 2000; Goidin *et al*, 2001). These PNA-positive melanoma cells actually exist in primary melanomas, and are associated with the degree of local invasion, which in turn governs the risk of metastases and poor clinical outcome (Berthier-Vergnes *et al*, 1993; Cochran *et al*, 1999). Taken together, these findings strongly suggest that PNA-positive cells within the primary tumour are those likely to invade dermis.

This study sought to identify candidate markers associated with the acquisition of dermal invasiveness. Oligonucleotide microarrays were used to discover genes that varied in expression level between two well-characterised invasive PNA-positive *vs* non-invasive PNA-negative melanoma cell clones, both selected *in vivo* from a non-aggressive parental cell line (Boukerche *et al*, 1994, 2007; Zebda *et al*, 1994, 1995; Béchetoille *et al*, 2000). TSPAN8, a member of the tetraspanin family, was thereby identified, confirmed as exclusively expressed by melanoma cells from a panel of invasive cell lines and functionally characterized as a novel mediator of cutaneous melanoma invasion.

## Materials and Methods

### Antibodies and reagents

Chemical reagents, primary and secondary antibodies used are listed in Supplementary Materials and Methods.

### Cell lines and cell culture

See Supplementary Materials and Methods for a detailed description of the cell lines used.

### DNA microarray hybridisation and data analysis

Extracted RNA from IC8 and T1C3 clones was further amplified using the AminoAllyl MessageAmp II aRNA amplification kit (Ambion/Applied Biosystems, Austin, TX, USA). Four independent dye-swap hybridisations (eight microarrays) were performed as we have described elsewhere (Bonin *et al*, 2009). Further experimental details, including microarray analysis, are described in Supplementary Materials and Methods.

### Semiquantitative RT–PCR

Semiquantitative PCR measurement of RELN, RNLS, CAPG, H2AFJ and FXYD5 expression was carried out in a Peltier thermal cycler (MJ Research Inc., Waltham, MA, USA). Primer sets and PCR conditions are detailed in Supplementary Materials and Methods.

### Quantitative real-time RT–PCR

Real-time PCR was performed on an Mx3000P real-time PCR system (Stratagene, La Jolla, CA, USA). Primer sets, PCR conditions and details of quantitation analysis are listed in Supplementary Materials and Methods. Three biological replicates, resulting from three different RNA extractions, were used for quantification analysis, and three technical replicates were analysed for each biological replicate.

### Western blot analysis

Subconfluent melanoma cell monolayers were washed with PBS and detached from the flask by a Cell Dissociation Buffer enzyme-free solution (Invitrogen Co., Carlsbad, CA, USA). Cells were lysed for 30 min at 4°C in RIPA buffer (50 mM Tris-HCl, pH 8.0, 150 mM NaCl, 0.5% sodium deoxycholate, 1% NP40, 0.1% SDS) and supplemented with protease inhibitors (Complete Mini; Roche Diagnostics, Mannheim, Germany). Protein concentration was assayed using the DC protein assay kit (BioRad Laboratories, Inc., Benicia, CA, USA). Equal amounts of protein (10 *μ*g) were separated on 10% SDS–polyacrylamide gel under non-reducing conditions, then transferred onto PVDF membrane. Membranes were blocked for 1 h at room temperature with 10 mM Tris-Buffer Saline, containing NaCl 300 mM, 1% Tween 20 and 10% non-fat milk, and then incubated overnight at 4°C with an antibody against human CAPG or TSPAN8. After washing, membranes were further incubated 1 h at room temperature with a peroxidase-conjugated goat anti-mouse antibody (Calbiochem, San Diego, CA, USA). Reactive bands were detected by enhanced chemiluminescence following the manufacturer's suggestions (ECL; GE Healthcare Life Sciences, Saclay, France), followed by a short exposure to X-ray film.

### Flow cytometry

Cell surface staining for FXYD5, TSPAN8, CD9, CD53, CD63, CD81, CD82, CD151, RELN and intracellular staining for BCL11A, CAPG, renalase and ID3 were carried out according to a procedure detailed in Supplementary Materials and Methods. The results are reported as mean fluorescence intensity of three independent experiments.

### Immunohistochemical analysis of normal skin and melanoma tissues

Formalin-fixed, paraffin-embedded melanocytic lesions, previously classified according to their tumour growth phase (RGP, VGP, metastasis), were screened for TSPAN8 protein expression by a biotin–streptavidin-amplified technique with an alkaline phosphatase kit (Dako-LSAB2-System, DAKO, Hamburg, Germany). Details of lesions and staining procedure can be found in Supplementary Materials and Methods.

### TSPAN8 gene knockdown with siRNA

T1C3 melanoma cells were seeded into six-well plates at 2 × 10^5^ cells per well, grown for 24 h in complete medium and then transfected with ON-TARGET plus Smart pool siRNA specific to TSPAN8 or scrambled siRNA-negative control (Dharmacon, Chicago, IL, USA) at a final concentration of 5 nM using Hyperfect transfection reagent (Qiagen, Courtaboeuf Cedex, France) according to the manufacturer's directions. To check TSPAN8 knockdown, cells were harvested 2, 3, 4, 5 and 6 days after siRNA transfection and analysed by RT–qPCR, western blot and flow cytometry as described above. Toxicity due to transfection was excluded, as assessed by flow cytometry using propidium iodide (Sigma, St Louis, MO, USA). Three days after transfection, cells were detached by accutase and used for cell viability, proliferation, migration and invasion assays, as described below.

### Cell proliferation and viability assays

BrdU incorporation was used to determine cell proliferation. Cell viability was determined by means of a colorimetric XTT assay. Experimental details can be found in Supplementary Materials and Methods.

### Migration assay

Migration of melanoma cells was assessed using a mechanical scratch wound assay as described elsewhere (Boukerche *et al*, 2007). The experimental procedure and the quantification analysis are detailed in Supplementary Materials and Methods.

### Three-dimensional spheroid invasion assay

Spheroid cell culture was performed using the hanging drop method, as described previously (Smalley *et al*, 2006). Further experimental details and invasive outgrowth quantification are given in Supplementary Materials and Methods. Two separate experiments were run in quadruplicate.

### Statistical analysis

The results were analysed for statistical significance using Student's *t*-test. Only *P*-values <0.05 were considered statistically significant (^*^P<0.05, ^**^P<0.01, ^***^P<0.001).

## Results

### Gene expression differences between invasive and non-invasive melanoma cells

To identify genes involved in the acquisition of an invasive potential, the gene expression profiles of invasive PNA-positive (T1C3 clone) and non-invasive PNA-negative (IC8 clone) melanoma cells, selected from the same parental cell line (Berthier-Vergnes *et al*, 1993; Zebda *et al*, 1994, 1995; Béchetoille *et al*, 2000; Goidin *et al*, 2001), were compared using oligonucleotide microarray. Of the 26 496 genes analysed (2% of probes spotted on microarray) 461 were differentially expressed between the two clones (fold-change ⩽2, Student's *t*-test *P*-value <0.01): 390 of these were downregulated in the invasive (T1C3 clone) melanoma cells, whereas only 71 were upregulated (Supplementary Tables S1, S2). Down- and upregulated genes implicated in major diseases and disorders or major molecular and cellular functions are listed in Supplementary Tables S3 and S4, respectively. As expected, the largest numbers of these regulated genes were those associated with cancer, inflammation and dermatological diseases (Supplementary Tables S1 and S3). Interestingly, the genes expressed most differentially between the two clones were involved in cell growth and death control, cell movement and invasion, cell assembly and organisation, cell-to-cell interaction, as well as DNA replication, recombination and repair (Supplementary Tables S1 and S4). Further analysis revealed that most of these genes critically regulated cell cycle, apoptosis, cell adhesion, cytoskeleton organisation, proteolysis and the intracellular signalling cascade.

Putative biological markers of dermal invasiveness are those genes that are overexpressed in invasive melanoma cells. Most are critically involved in the regulation of cell movement and invasion, as well as proliferation (Supplementary Tables S1 and S4). Some of them encode for proteins involved in cell adhesion and actin-based motility, including ICAM2, *α*2-actin (ACTA2), autocrine motility factor/glucose phosphate isomerase, extracellular matrix serine protease (RELN), pigment epithelium-derived factor (SERPINF1), a tetraspanin family member (TSPAN8) and CAPG (capping protein-actin filament-gelsolin-like) (Supplementary Table S4), highlighting the well-known importance of tumour cell interaction with the extracellular matrix during invasion. In line with this, integrin signalling emerged as one of the top relevant biological pathways (Ingenuity score=2.65E–03). Furthermore, ERK/MAPK (Ingenuity score=9.38E–03) was the second top signalling pathway, known to have a key role in the pathogenesis of cutaneous melanoma (Meier *et al*, 2005), thus confirming the reliability of our study design.

### Validation of microarray candidate genes

Transcripts that are up- rather than downregulated in invasive cells may have potential prognostic and therapeutic relevance. We therefore focused on the genes that exhibited the greatest fold-change (>2.8) (Supplementary Table S2). Ingenuity pathways analysis highlighted that cancer and cellular movement/invasion were the functional classes most consistently found to be enriched in this set of genes (Supplementary Tables S3 and S4). We thus excluded genes unrelated to cancer and those related to cancer, but not to cellular movement/invasion (Supplementary Tables S3 and S4). Among them, SERPINF1 was excluded because it encodes the secreted epithelium-derived factor (PEDF), also found in normal melanocytes (Orgaz *et al*, 2009). RNLS encoding renalase, a recently discovered secreted amine oxidase involved in kidney disease, was included, as amine oxidases are well-known regulators for cancer progression. H2AFJ, encoding a member of the histone H2A family, was also included, as a previous cDNA microarray study of a melanoma model had found it to be upregulated in metastatic cells (de Wit *et al*, 2005). Finally, seven genes were selected for further validation: BCL11A, CAPG, RELN, TSPAN8, ID3, FXYD5 and RNLS.

BCL11A, the B-cell associated transcription factor leukaemia 11A gene encoding a zinc-finger protein, acts as a transcriptional repressor critical to lymphoma malignancy (Satterwhite *et al*, 2001). CAPG encodes an actin filament end capping of the gelsolin family crucial for the control of cell migration or invasion in a variety of cancer cells (Renz *et al*, 2008). RELN encodes reelin, a matrix serine protease regulating neural cell migration and tumour progression (Perrone *et al*, 2007). TSPAN8 encodes tetraspanin 8, also known as CO-029, a transmembrane protein overexpressed in numerous carcinomas (Boucheix *et al*, 2001; Hemler, 2005; Zöller, 2009). ID3 gene encodes a protein DNA-binding protein inhibitor, overexpressed in several carcinomas and reported to be involved in tumour growth, invasiveness, metastasis and angiogenesis (Lasorella *et al*, 2001). FXYD5 encodes dysadherin, a cancer-associated glycoprotein that promotes tumour metastasis by downregulating E-cadherin in numerous human carcinomas (Ino *et al*, 2002).

**Figure 1:**
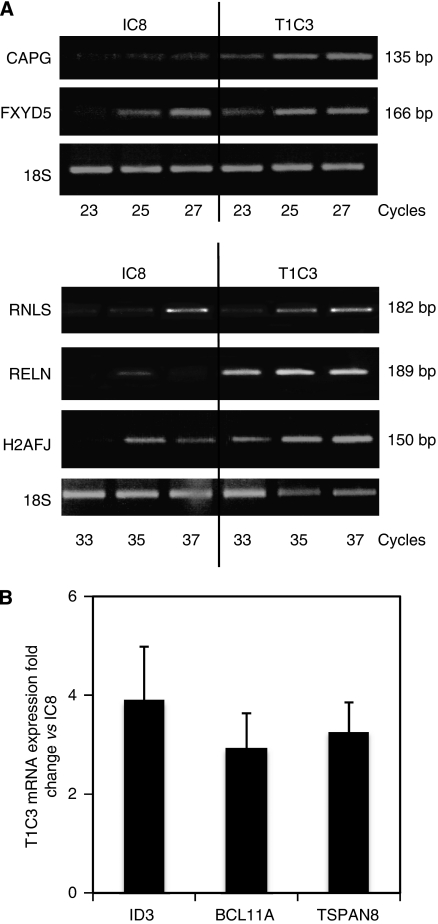
Reverse transcription–polymerase chain reaction (RT–PCR) analysis of eight selected genes differentially expressed in invasive (T1C3) and non-invasive (IC8) clones, according to microarray analysis. (**A**) Semiquantitative PCR was performed with total RNA isolated from both clones. PCR products were loaded on a 1.2% agarose gel and stained with ethidium bromide. The expected length of PCR products is indicated on the right. The data are representative of three experiments. (**B**) Quantitative RT–PCR analysis of three genes in T1C3 cells compared with IC8 cells. Copy number was first normalised to 18SRNA levels and expressed as the fold-change over IC8 cells. The graph depicts the mean of these fold-changes ±s.d. of three independent amplifications.

To further validate microarray data, transcript levels of the selected genes were measured by RT–PCR. Semiquantitative RT–PCR results for CAPG, FXYD5, RNLS, RELN and H2AFJ (Figure 1A) and real-time quantitative PCR results for ID3, BCL11A and TSPAN8 (Figure 1B) were consistent with the microarray data. The upregulation of these genes in T1C3 melanoma cells was also confirmed in two other experiments, using RNA extracts from both clones cultivated for different numbers of passages (data not shown).

### TSPAN8 and CAPG proteins were highly expressed in T1C3 melanoma cells

**Figure 2:**
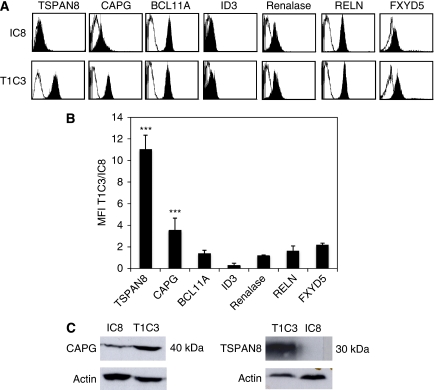
TSPAN8 and CAPG are strongly upregulated in T1C3 invasive melanoma cells. Invasive (T1C3) and non-invasive (IC8) melanoma cells were cell surface stained with mAbs directed against TSPAN8, RELN and FXYD5, and intracellularly stained with anti-CAPG, BCL11A, ID3 and renalase. (**A**) Data from a representative experiment showing flow cytometry profile of IC8 and T1C3 melanoma cells stained with the indicated mAbs. Filled histograms represent specific and open histograms isotype-matched control antibodies. (**B**) Results for a given protein are expressed as the following ratio: MFI in T1C3/MFI in IC8 cells ±s.d. of three independent experiments. Statistical significance was assessed using Student's *t*-test: ^***^P<0.001. (**C**) Total cell lysates from IC8 and T1C3 were subjected to western blot analysis with antibodies specific for CAPG and TSPAN8, actin serving as a loading control.

To validate mRNA expression, the expression levels of the selected gene products on IC8 and T1C3 cells were compared by flow cytometry. Melanoma cells were surface stained for TSPAN8, RELN and FXYD5 or intracellularly stained for renalase, CAPG, BCL11A and ID3. As shown in a representative experiment (Figure 2A), BCL11A, ID3, renalase and RELN were expressed at similar levels in both clones. In contrast, TSPAN8, CAPG and FXYD5 expressions were markedly higher in T1C3 than IC8 cells. Figure 2B summarises the results from three independent experiments, confirming that only TSPAN8 and CAPG were significantly overexpressed in T1C3 compared with IC8 cells. Indeed, T1C3 cells exhibited an approximately 11- and 4-fold cell surface expression of TSPAN8 and CAPG, respectively, than did IC8 cells. The differential expression of these two proteins was further confirmed by western blot analysis (Figure 2C). It should be noted, however, that TSPAN8 was so weakly expressed in IC8 melanoma cells that no band could be visualised (Figure 2C). Taken together, these results show that TSPAN8 and CAPG were more highly expressed at both mRNA and protein levels in T1C3 than in IC8 cells.

### TSPAN8 was the only tetraspanin in which overexpression correlated with the invasive phenotype

**Figure 3:**
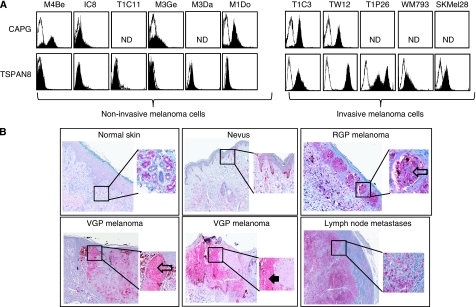
TSPAN8 is expressed by invasive melanoma cells in culture and in melanoma lesions. (**A**) Non-invasive (M4Be, M3Ge, M3Da, M1Do cell lines and IC8, T1C11 clones) and invasive melanoma cells (WM793, SKMel28 cell lines and T1C3, TW12, T1P26 clones) were cell surface stained with antibodies directed against TSPAN8 and CAPG. Filled histograms represent specific and open histograms isotype-matched control antibodies. Results are representative of three independent experiments. (**B**) Representative immunohistochemical expression of TSPAN8 immunostaining in normal skin, benign nevus, RGP and VGP melanomas, and lymph node metastases. Note negative staining of cells from normal skin, except for eccrine glands, which was useful as an internal positive control. The square represents the area of magnification shown in the inset. Open arrows pointing at positive stained junctional nests of melanocytes. Black arrow pointing at a dermal nest of stained cells.

We then investigated the expression of TSPAN8 and CAPG protein on another seven melanoma cell lines/clones, in addition to the two clones, to find any correlation with the invasive potential of melanoma cells. These cell lines and clones, widely used as a metastasis melanoma model (Doré *et al*, 1994; Zebda *et al*, 1994, 1995; Béchetoille *et al*, 2000; Goidin *et al*, 2001; Boukerche *et al*, 2007), were classified into two groups: (a) PNA-moderate or -negative cells with low or no invasive potential: M4Be, T1C11, M3Ge, M3Da and M1Do; and (b) PNA-positive cells with a high invasive potential: TW12 and T1P26. Flow cytometry demonstrated that CAPG was expressed not only by cells belonging to the non-invasive group, especially the M4Be, M3Ge, M1Do cell lines, but also by TW12 clone from the invasive group (Figure 3A). In contrast, TSPAN8 was exclusively expressed by cells belonging to the invasive group. The correlation between TSPAN8 expression and aggressiveness was not restricted to our melanoma model, as SKMel28 and WM793 melanoma cell lines, chosen because they are able to invade dermis in human skin reconstructs (Hsu *et al*, 2008; Lasithiotakis *et al*, 2008), also expressed TSPAN8 on their cell surface (Figure 3A).

Because previous research indicated that tetraspanins may contribute to the metastatic process (Boucheix *et al*, 2001; Hemler, 2005; Zöller, 2009), we next analysed the cell surface expression profiles of other tetraspanins (CD9, CD53, CD63, CD81, CD82 and CD151) on invasive and non-invasive melanoma cells. As shown in a representative experiment (Supplementary Figure S1), TSPAN8 was the only tetraspanin able to distinguish between invasive and non-invasive cells. Importantly, TSPAN8 was not expressed on normal melanocytes (Supplementary Figure S1). Therefore, the cell surface expression of TSPAN8, but not that of CAPG, was associated with the invasive behaviour of a panel of melanoma cells in culture.

### TSPAN8 was expressed in primary melanomas and in lymph node metastases, but not in normal epidermis

Despite the observation of TSPAN8 overexpression in various carcinomas (Boucheix *et al*, 2001; Hemler, 2005; Zöller, 2009), no information is available for human cutaneous melanoma. We therefore further examined *in situ* TSPAN8 expression in melanocytic lesions. Immunohistochemistry was performed on archival formalin-fixed, paraffin-embedded sections of tissue ranging from normal skin to benign tumour, as well as invasive and metastatic melanocytic lesions. The samples covered all major types of melanocytic proliferation, including 16 benign nevi (8 congenital, 8 compound), 13 primary melanomas in RGP, 35 primary melanomas that had entered VGP and 6 melanoma metastases to lymph nodes. Normal skin was not stained by anti-Tspan8 and half of benign lesions showed a weak staining in few nevus cells (Figure 3B; Supplementary Table S5). In contrast, melanoma cells stained strongly positive for TSPAN8 in primary melanomas and in lymph nodes (Figure 3B): immunoreactivity was observed in 8 of the 13 RGP lesions, in 10 of the 35 VGP lesions and in 2 of the 6 lymph node metastases (Supplementary Table S5). Numerous positive cell nests were located near the dermal–epidermal junction in both the intraepidermal and the dermal components of the primary lesions (Figure 3B). At higher magnification, cytoplasm and membrane staining of varying intensity could be observed in primary and metastatic melanoma lesions.

### Transient endogenous TSPAN8 knockdown did not significantly impair cell survival, proliferation or cell migration

**Figure 4:**
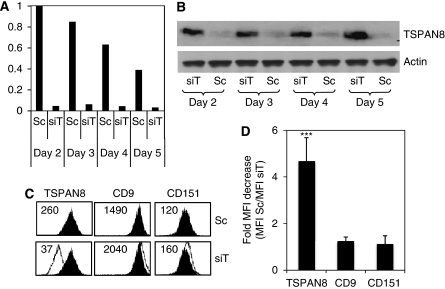
Knocking down of endogenous TSPAN8 expression is efficient and specific. T1C3 melanoma cells were transfected with either TSPAN8-specific (siT) or control scramble siRNAs (Sc). (**A**) Total RNA was extracted at the indicated time after transfection and transcript levels of TSPAN8 were detected by qRT–PCR. Relative mRNA expression of TSPAN8 in different cell lines was normalised to the signal intensity of 18S RNA as an internal control. A representative experiment of three independent transfection assays is shown. (**B**) Cell lysates from siRNA-treated cells were subjected to western blot analysis with antibodies specific for TSPAN8 or actin at the indicated days after transfection. (**C**) Melanoma cells were cell surface stained using the indicated mAbs at day 3 after transfection. Data from a representative experiment showing flow cytometry profile of cells transfected with scramble (black) or TSPAN8 (white) siRNAs. Mean fluorescence intensity is reported in each histogram. (**D**) Results for a given tetraspanin are expressed as MFI of T1C3 transfected with scramble siRNA/MFI of T1C3 transfected with TSPAN8 siRNA, and are the mean±s.d. of six independent experiments. Statistical significance was assessed using Student's *t*-test: ^***^P<0.001.

Although several tetraspanins have been implicated as regulators of cell proliferation, migration and invasion of tumour cells (Boucheix *et al*, 2001; Hemler, 2005; Zöller, 2009), the function of TSPAN8 is still unknown. To examine whether it was also involved in these cellular processes, we first knocked down endogenous TSPAN8 by transient transfection of invasive T1C3 cells with specific siRNA. As shown in Figure 4, the TSPAN8 silencing effect was efficient: a strong reduction of both TSPAN8 mRNA and protein expression was confirmed with TSPAN8-targetting siRNA as early as 2 days after transfection and persisted for up to 5 days, as assessed by qRT–PCR (Figure 4A), western blot (Figure 4B) and flow cytometry (Figure 4C and D). Mean silencing of TSPAN8 transcripts averaged 89±4.6 and 72±5.8% at 2 and 5 days post-transfection, respectively (*n*=3), without significant impact on the expression of other two major tetraspanins expressed by both melanoma clones: CD9 and CD151 (Figure 4C and D), thus demonstrating the specificity of the silencing effect.

**Figure 5:**
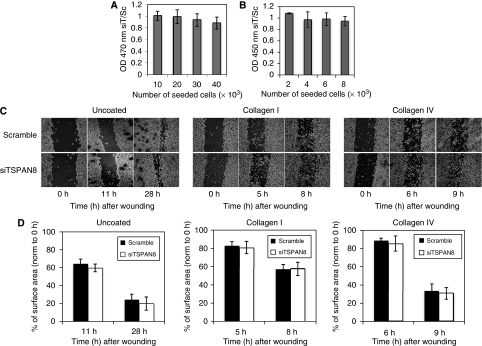
Endogenous TSPAN8 silencing has no impact on cell survival, proliferation and migration. T1C3 melanoma cells were transfected with siRNA targeting TSPAN8 (siTSPAN8) or siRNA scramble (scramble). (**A**) Cell viability determined by XTT assay 3 days post-siRNA treatment of T1C3 melanoma cells. Data are expressed as mean OD 450 nm of siTSPAN8-treated cells/mean OD 450 nm in scramble siRNA-treated cells, ±s.d. of three independent experiments. (**B**) Cell proliferation determined by BrdU assay 3 days post-siRNA treatment of T1C3 melanoma cells. Data are expressed as in A. (**C**) Cells grown to confluence in uncoated, collagen I- or collagen IV-coated six-well plates were wounded by creating a scratch across the monolayer culture 3 days after transfection. Representative photographs showing this region were taken directly following injury (0 h) and at various time later using a Sony DXC-390 digital camera under an inverted phase microscope (Zeiss LSM510, Zeiss Inc., Thornwood, NY, USA). (**D**) Quantification of wound closure depicted in C. Data are expressed as percentage of the initial wound size and set to 100% at 0 h. No statistically significant difference was observed at any time point (*P*>0.05).

We next sought to determine the role and significance of endogenous TSPAN8 silencing on melanoma cell viability and proliferation. As shown in Figure 5A, the reduced expression of TSPAN8 in invasive melanoma cells did not alter cell viability, as evaluated by XTT assay. Likewise, the cell proliferation measured by BrdU ELISA assay did not reveal any significant disturbance (Figure 5B). Furthermore, TSPAN8 silencing was not accompanied by changes in cell morphology (not shown).

The correlation between TSPAN8 expression and the invasive phenotype led us to examine whether this protein was critical to the migratory behaviour of melanoma cells. As illustrated in a representative experiment (Figure 5C), 28 h after a wound had been generated in a confluent cell monolayer, closure was almost complete in both silenced and scrambled T1C3 cells. Quantitative analysis of the areas recolonised by the cells in three independent experiments confirmed that the reduced expression of TSPAN8 did not significantly alter the migration rate (Figure 5D). As additional controls, T1C3 cells displaying endogenous TSPAN8 and IC8 cells devoid of TSPAN8 were also examined and found to exhibit similar migratory behaviour (data not shown).

Because several studies have reported tetraspanins involvement in cell–matrix interactions, scratch wound assay was also performed on cells plated on the two main components of the basement membrane and dermis (type IV- or type I-collagen, respectively) and on a reconstituted basement membrane (matrigel). No significant impairment in wound closure was detected between melanoma cells transfected with TSPAN8-specific or scramble siRNAs in the presence of either type of collagen (Figure 5C and D), as well as on matrigel (data not shown).

### Transient endogenous TSPAN8 knockdown reduced the invasive outgrowth of melanoma cells embedded in matrigel

**Figure 6:**
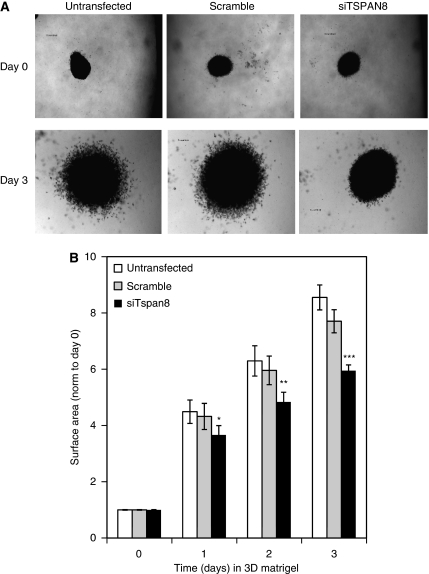
Endogenous TSPAN8 silencing reduces melanoma cell invasion. T1C3 melanoma cells transfected with siRNA targeting TSPAN8 (siTSPAN8), siRNA scramble (scramble) or untransfected were seeded 2 days later into a thick layer of matrigel. (**A**) Micrographs depict melanoma spheroids embedded in three-dimensional matrigel immediately after seeding (day 0) and at day 3. (**B**) Invasive outgrowth was quantified by calculating the ratio between the area spheroid after a given incubation period to the original spheroid area at 0 h. Spheroid areas at day 0 were set to 1. Bars represent the means±s.d. of the spheroid area from quadruplicates (*P*<0.001). Statistical significance was assessed using Student's *t*-test: ^*^P<0.05; ^**^P<0.01; ^***^P<0.001.

It is now acknowledged that three-dimensional (3D) spheroid culture more accurately mimics the tumour microenvironment than two-dimensional (2D) monolayer culture (Smalley *et al*, 2006; Pampaloni *et al*, 2007). Therefore, T1C3 melanoma cells were grown as cellular spheroids in a 3D matrix, a well-known model system previously shown to adequately reflect melanoma stage (RGP, VGP or metastatic) (Smalley *et al*, 2006). As illustrated in a representative experiment (Figure 6A), the multicellular aggregates of melanoma cells formed a cell-dense region, analogous to the core of the primary tumour. Importantly, T1C3 transfected with scramble or TSPAN8-specific siRNA and untransfected cells all formed compact spheroids that increased in size over 3 days, corroborating the finding that TSPAN8 is not required for melanoma growth. However, T1C3 cells transfected or not with scramble siRNA exhibited spheroids that progressively infiltrated the surrounding matrigel matrices, mimicking invading cells (Figure 6A). In contrast, TSPAN8-knockdown spheroids showed restrictive invasive movement away from the spheroid edge (Figure 6A). Quantitation of invasive outgrowth indicated that the total spheroid surface area of TSPAN8 siRNA-transfected cells significantly decreased nearly 1.8-fold compared with scramble siRNA-transfected cells or untransfected cells at day 3 (Figure 6B). Altogether, these results indicate that TSPAN8 has a role in the invasive behaviour of melanoma cells.

## Discussion

The alterations that allow progressive invasion into the dermis remain ill defined, largely due to the limited availability of primary tissues, their heterogeneous nature, the interindividual genetic variability of tissue specimens and derived cell lines, and the lack of suitable experimental models. There have, therefore, been few true advances towards the development of reliable diagnostic and prognostic biomarkers in cutaneous melanoma (Ohsie *et al*, 2008). We therefore compared global gene expression profiles of two clonal subpopulations selected from the same human melanoma cell line, but differing in ability to cross the dermal–epidermal junction in skin reconstructs (Béchetoille *et al*, 2000; Goidin *et al*, 2001). The study spotlighted TSPAN8, a member of the tetraspanin family, showing it to be strongly expressed, at both mRNA and protein levels, by invasive melanoma cells as compared with normal melanocytes or non-invasive melanoma cells. Its cell surface expression correlated with the invasive phenotype of a panel of melanoma cell lines. Consistent with the *in vitro* findings, TSPAN8 was also expressed by melanoma cells in primary tumours and lymph node metastases, but not in healthy epidermis. More importantly, the functional role of TSPAN8 was demonstrated by silencing endogenous TSPAN8 with siRNA, which reduces invasive outgrowth from tumour spheroids within matrigel, without impact on the cell proliferation and survival. To the best of our knowledge, this is the first study to report that TSPAN8 is likely to have a critical role in cutaneous melanoma invasion.

The transition from RGP to VGP is considered to be the high point of change in gene expression patterns during melanoma progression (Haqq *et al*, 2005; Smith *et al*, 2005; Jaeger *et al*, 2007; Jensen *et al*, 2007; Riker *et al*, 2008), consistent with tumour thickness being one of the strongest predictors of metastatic disease and adverse clinical outcome (Miller and Mihm, 2006; Ohsie *et al*, 2008). This study used well-characterised melanoma cell populations, previously selected from a parental cell line (Bailly and Doré, 1991) and differing in their ability, to invade dermis in human skin reconstructs (Béchetoille *et al*, 2000; Goidin *et al*, 2001). Notably, PNA-positive melanoma cells (T1C3 clone) grew in a pattern resembling RGP lesions, whereas PNA-negative cells (IC8 clone) displayed a VGP growth pattern: the former invaded and proliferated deep in the dermis, whereas IC8 cells remained in the epidermis (Béchetoille *et al*, 2000; Goidin *et al*, 2001). A large-scale oligonucleotide microarray comparative transcriptome analysis of PNA-positive and PNA-negative melanoma cells was performed. These two cell populations were unlikely to show the genetic heterogeneity that can complicate the interpretation of microarray data. Indeed, few genes were differentially expressed (2%), 390 of which were downregulated and only 71 upregulated in the invasive melanoma cells. Interestingly, the microarray data revealed less upregulated than downregulated genes in the invasive melanoma cells. This is consistent with data from other studies (Haqq *et al*, 2005; Winnepenninckx *et al*, 2006; Jaeger *et al*, 2007; Ryu *et al*, 2007), in which the switch from RGP to VGP was mainly accompanied by loss of gene expression, validating our experimental model.

TSPAN8 belongs to a large family of integral membrane proteins, the tetraspanins, characterised by the presence of four highly conserved transmembrane domains. Also known as C0-029 in humans and D6.1 in rats, TSPAN8 was first identified as a tumour-associated antigen present at high levels in several types of human carcinoma and sarcoma (Boucheix *et al*, 2001; Zöller, 2009). Its overexpression correlates with gastrointestinal and pancreatic cancer progression, thus representing a marker of poor prognosis (Boucheix *et al*, 2001; Zöller, 2009). Here, we show for the first time that TSPAN8 is strongly expressed in human cutaneous melanoma. Interestingly, TSPAN8 was not constitutively expressed by normal melanocytes, in contrast to nevocytes and melanoma cells from primary and metastatic lesions. This suggests that the expression of TSPAN8 occurs very early during tumour development.

It was initially thought that invasion and dissemination occurred during the VGP. However, gene profiling studies by several research teams strongly suggest that invasive potential may exist early in melanoma development (Hoek *et al*, 2004; Haqq *et al*, 2005; Talantov *et al*, 2005; Riker *et al*, 2008). In support of this theory, Eyles *et al* (2010) recently demonstrated, in a mouse melanoma model, that melanoma cells emigrate to remote organs very early during the development of the primary tumour. Here, we found a higher frequency of TSPAN8 expression in early (RGP) than later stages of melanoma progression (VGP, metastatic). Although further studies on larger series of melanocytic lesions will be necessary to confirm these findings, the idea of early spread led us to speculate that TSPAN8 might identify metastatic cells arising from early primary tumours.

The tetraspanins have a unique ability to associate laterally with one another and to cluster dynamically with other transmembrane and signal-transducing partners, notably, integrins (Boucheix *et al*, 2001; Hemler, 2005; Zöller, 2009). Through these multimolecular complexes, several tetraspanins are thought to mediate steps of the metastatic cascade. Claas *et al* (2005) reported that D6.1A associates with tetraspanins CD151, CD9, CD81, integrins *α*3*β*1 and *α*6*β*4, and also with other partners, including EWI-F and EpCAM, on rat carcinoma cells. Le Naour *et al* (2006) found that TSPAN8 was detected by mass spectrometry only in CD9 complexes collected from high invasive colon carcinoma. Furthermore, several tetraspanins may also associate with non-protein partners, such as gangliosides (Kawakami *et al*, 2002). We found here that TSPAN8 was the only tetraspanin able to distinguish invasive from non-invasive melanoma cells. Because PNA-positive cells display a peculiar cell surface profile of integrins (Boukerche *et al*, 1994) and gangliosides (Zebda *et al*, 1995), identifying TSPAN8 partners in invasive human melanoma cells is therefore essential for understanding the functions conferred by TSPAN8, and is currently under investigation.

Very little is known about the mechanisms of TSPAN8-mediated metastasis promotion. It is thought that TSPAN8 contributes to the cell motility of metastatic carcinoma cell lines, mostly through its association with *α*6*β*4 and CD151 following PMA treatment (Herlevsen *et al*, 2003; Gesierich *et al*, 2005), whereas association with *α*3*β*1 and *α*6*β*1 promotes haematogenic spread of tumour cells (Claas *et al*, 1998; Kanetaka *et al*, 2003). An interesting finding in this study is that siRNA TSPAN8 knockdown reduced the invasive outgrowth of melanoma spheroids embedded in matrigel. It seems unlikely that this was caused by a decrease in cell survival or growth rate, as TSPAN8 silencing did not affect viability or proliferation. Our observations are in line with previous studies showing that ectopic TSPAN8 expression in low-metastatic carcinoma cells did not confer any advantage for cell proliferation but led to a high metastatic potential (Claas *et al*, 1998; Kanetaka *et al*, 2003; Zhou *et al*, 2008).

Three-dimensional models of melanoma culture represent a technical approach that adequately reflects the clinical melanoma tumour stage: cell lines derived from VGP, but not RGP, melanoma lesions invade 3D matrices (Smalley *et al*, 2006). Therefore, we here used melanoma spheroids embedded in a basement-membrane-like extract (matrigel) to mimic the invasive movement of melanoma cells through the basement membrane. Although TSPAN8 silencing reduced the invasive outgrowth of melanoma cells embedded in matrigel, it did not impair their ability to close an artificial gap created over a confluent monolayer, even when cells were grown on plates coated with collagen-IV (the main basement membrane component), collagen-I (the most abundant component of the dermis) or a recontituted basement membrane: matrigel. These unexpected findings were consistent with the notion that the invasive capacity of tumour cells studied in 3D systems is not necessarily correlated with efficient migration on planar 2D surfaces (Torka *et al*, 2006). This is not surprising, given that the engagement of integrins (the main partners of tetraspanins) with matrix proteins was differentially regulated in the 2D relative to the 3D context (Cukierman *et al*, 2001). Indeed, the 2D- and 3D-matrix–integrin adhesion structures strongly differed in composition, thus recruiting of distinct cytoskeleton and cytoplasmic proteins, which control signalling pathways, and therefore, the mode of cell migration (Doyle *et al*, 2009). Given that the migratory/invasive behaviour of melanoma cells cultured in a 3D environment required a Rho GTPase protein (Klein and Aplin, 2009), further studies will be needed to investigate whether TSPAN8-mediated invasion is dependent on Rho signalling.

In conclusion, the present findings highlight several new aspects of the role of TSPAN8 in the initial steps of human melanoma progression. If the present *in vitro* findings have any physiological relevance, it is thus conceivable that TSPAN8 expression might give melanoma cells the ability to cross the cutaneous basement membrane, an early event leading to dermal invasion and progression to metastatic disease. Although much is yet to be learned regarding the clinical relevance of its function, we postulate that TSPAN8 could be a promising new therapeutic target in anti-invasive therapies for cutaneous melanoma.
