# Supplementary material for: Gene expression profiles of human melanoma cells with different invasive potential reveal TSPAN8 as a novel mediator of invasion
Source: Br J Cancer. 2010 Nov 16;104(1):155–65. doi: 10.1038/sj.bjc.6605994 (PMC3039798; doi:10.1038/sj.bjc.6605994)
Supplement: Supplementary Figure S1 [file 6605994x1.doc]

**Figure S1.** TSPAN8 expression correlates with the invasive phenotype of melanoma cells.


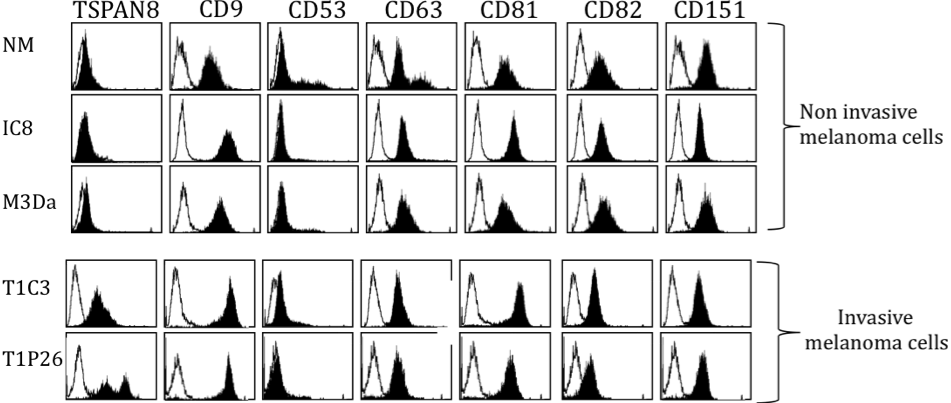


Normal melanocytes (NM), melanoma cells from non-invasive M3Da cell line and IC8 clone and from invasive clones (T1C3, T1P26), were cell-surface stained with antibodies directed against a panel of the indicated tetraspanins. Filled histograms represent specific and open histograms isotype-matched control antibodies. Results are representative of three independent experiments.
