# Supplementary material for: Gene expression profiles of human melanoma cells with different invasive potential reveal TSPAN8 as a novel mediator of invasion
Source: Br J Cancer. 2010 Nov 16;104(1):155–65. doi: 10.1038/sj.bjc.6605994 (PMC3039798; doi:10.1038/sj.bjc.6605994)
Supplement: Supplementary Table S1 [file 6605994x2.doc]

**Supplementary Table S1. Up-regulated(up) and down-regulated (down) genes in invasive melanoma cells versus non invasive melanoma cells**

| **461 Genes Differentially Regulated** (listed in supplementary Table S2) | | | | | |
| --- | --- | --- | --- | --- | --- |
| **71 Genes Up-Regulated** | | | **390 Genes Down-Regulated** | | |
| **Diseases and disorders** | | **up** | **down** | **Total** | **Genes listed in** |
| Cancer | | 30 | 151 | 181 | Supplementary Table S3 |
| Genetic disorders | | 28 | 136 | 164 |
| Inflammation | | 11 | 28 | 80 |
| Metabolic Disease | | 6 | 29 | 35 |
| Dermatological Disease | | 6 | 38 | 44 |
|  | Melanoma | 0 | 21 | 21 |
|  | Dermatological Disorders | 5 | 20 | 25 |
|  | Basal_Cell Carcinoma | 1 | 3 | 4 |
|  | Hyperplasia | 2 | 3 | 5 |
|  | Skin Tumors | 1 | 13 | 14 |
| **Molecular and Cellular Functions** | | **up** | **down** | **Total** | **Genes listed in** |
| Cellular Growth and Cell Death | | 23 | 171 | 194 | Supplementary Table S4 |
| Cellular Movement and Invasion | | 42 | 143 | 186 |
| Cellular Assembly and Organisation | | 10 | 65 | 75 |
| Cell to cell Signaling and Interaction | | 10 | 51 | 61 |
| DNA Replication, recombination and Repair | | 5 | 29 | 34 |

Genes displaying greater than 2 fold differential expression in invasive melanoma cells (T1C3 clone) and non invasive melanoma cells (IC8 clone) were functionally annotated to their implication in major diseases and disorders and in molecular and cellular functions using Ingenuity Pathway Analysis. Up-regulated genes (up) and down-regulated (down) in invasive melanoma cells versus non invasive melanoma cells. (Student’s t-test p value lower than 0.001).
