# Supplementary material for: Gene expression profiles of human melanoma cells with different invasive potential reveal TSPAN8 as a novel mediator of invasion
Source: Br J Cancer. 2010 Nov 16;104(1):155–65. doi: 10.1038/sj.bjc.6605994 (PMC3039798; doi:10.1038/sj.bjc.6605994)
Supplement: Supplementary Table S2 [file 6605994x3.doc]

**Suplementary Table S2.** List of the 461 genes differentially regulated between invasive melanoma cells (T1C3 clone) and non invasive melanoma cells (IC8 clone), and displaying at least 2 fold differential expression after initial normalization of microarray data (Student’s t-test p value lower than 0.001).

| Differentially Regulated Genes | | | |
| --- | --- | --- | --- |
| Symbol | Genebank | Fold Change (ratio T1C3/IC8) | Student’s t-test p-value |
| SERPINF1 | NM_002615 | 19.251 | 3,78E-04 |
| BCL11A | NM_022893 | 17.664 | 1,14E-05 |
| RNLS | NM_018363 | 16.745 | 1,15E-04 |
| MGST1 | NM_020300 | 14.997 | 3,39E-04 |
| H2AFJ | NM_177925 | 8.734 | 5,93E-04 |
| CAPG | NM_001747 | 7.435 | 4,29E-04 |
| RELN | NM_005045 | 6.782 | 1,64E-04 |
| SULT1A2 | NM_001054 | 6.080 | 1,11E-04 |
| TSC22D4 | NM_030935 | 4.756 | 4,39E-04 |
| PRDX2 | NM_005809 | 4.664 | 9,29E-05 |
| BAAT | NM_001701 | 4.393 | 1,08E-04 |
| DENND2D | NM_024901 | 4.242 | 1,50E-03 |
| HERPUD1 | NM_014685 | 4.010 | 5,55E-05 |
| IFITM2 | NM_006435 | 3.841 | 3,52E-04 |
| ZNF45 | NM_003425 | 3.780 | 5,55E-05 |
| TSPAN8 | NM_004616 | 3.408 | 7,12E-03 |
| ID3 | NM_002167 | 3.337 | 2,05E-03 |
| FAM164C | NM_024643 | 3.114 | 3,64E-04 |
| PNOC | NM_006228 | 3.072 | 4,89E-04 |
| NDUFA11 | NM_175614 | 2.962 | 5,40E-04 |
| FAM174B | BC031970 | 2.843 | 1,55E-04 |
| FXYD5 | NM_014164 | 2.821 | 1,88E-04 |
| MRPL4 | NM_015956 | 2.738 | 6,26E-04 |
| SLC45A2 | NM_016180 | 2.672 | 4,34E-04 |
| STOML3 | NM_145286 | 2.672 | 1,39E-03 |
| ODC1 | NM_002539 | 2.651 | 3,08E-04 |
| LONP1 | NM_004793 | 2.605 | 3,89E-04 |
| GPI | NM_000175 | 2.582 | 2,78E-04 |
| ASNA1 | NM_004317 | 2.565 | 2,04E-04 |
| PCBD1 | NM_000281 | 2.553 | 2,73E-03 |
| STOX1 | NM_152709 | 2.549 | 1,15E-04 |
| NDUFB7 | NM_004146 | 2.447 | 6,69E-04 |
| CD79B | NM_000626 | 2.432 | 2,53E-04 |
| CES1 | NM_001266 | 2.430 | 4,07E-03 |
| FRZB | NM_001463 | 2.400 | 1,22E-03 |
| ABCC5 | NM_005688 | 2.366 | 1,88E-04 |
| PUS7L | NM_031292 | 2.349 | 1,33E-04 |
| TYRP1 | NM_000550 | 2.320 | 6,10E-03 |
| TP53TG1 | NM_007233 | 2.294 | 2,11E-03 |
| HSD3B2 | NM_000198 | 2.274 | 4,89E-04 |
| CNN2 | NM_004368 | 2.242 | 3,23E-04 |
| HAND1 | NM_004821 | 2.235 | 2,04E-04 |
| KIAA1804 | NM_032435 | 2.230 | 1,39E-03 |
| PPP2R1A | NM_014225 | 2.189 | 2,92E-04 |
| CPT1C | NM_152359 | 2.179 | 1,78E-03 |
| DNAH10 | AJ132089 | 2.158 | 7,23E-04 |
| HERC6 | NM_017912 | 2.154 | 9,99E-03 |
| SCLY | NM_016510 | 2.145 | 1,16E-03 |
| GUK1 | NM_000858 | 2.135 | 6,04E-04 |
| ACTA2 | NM_001613 | 2.128 | 1,59E-03 |
| PGM1 | NM_002633 | 2.113 | 6,93E-04 |
| MRVI1 | NM_006069 | 2.109 | 2,23E-03 |
| COX7B | NM_001866 | 2.104 | 2,56E-04 |
| EBPL | NM_032565 | 2.103 | 2,10E-03 |
| PPIB | NM_000942 | 2.098 | 2,83E-03 |
| PAM | NM_000919 | 2.097 | 1,75E-03 |
| ISOC2 | NM_024710 | 2.087 | 2,78E-04 |
| PDLIM4 | NM_003687 | 2.079 | 1,57E-03 |
| CYC1 | NM_001916 | 2.070 | 9,55E-04 |
| PLCD1 | NM_006225 | 2.067 | 8,23E-04 |
| DNAJB1 | NM_006145 | 2.066 | 1,00E-03 |
| ID1 | NM_002165 | 2.062 | 3,72E-04 |
| SVEP1 | NM_153366 | 2.046 | 9,51E-04 |
| ICAM2 | NM_000873 | 2.041 | 5,59E-03 |
| HPGD | NM_000860 | 2.035 | 7,11E-04 |
| UQCRQ | NM_014402 | 2.030 | 2,07E-03 |
| ACO2 | NM_001098 | 2.025 | 2,78E-04 |
| THSD7A | AB023177 | 2.019 | 3,45E-03 |
| RPS15 | NM_001018 | 2.018 | 2,85E-03 |
| CD53 | NM_000560 | 2.011 | 4,31E-04 |
| CCDC87 | NM_018219 | 2.002 | 3,40E-04 |
| KPNA2 | NM_002266 | -2.000 | 3,51E-03 |
| MTHFD1 | NM_005956 | -2.002 | 2,88E-04 |
| PSMB5 | NM_002797 | -2.004 | 1,54E-03 |
| C14ORF1 | NM_007176 | -2.005 | 2,73E-03 |
| KCNC1 | NM_004976 | -2.005 | 2,88E-04 |
| SH3BGRL | NM_003022 | -2.005 | 3,32E-03 |
| ZDHHC7 | NM_017740 | -2.006 | 6,18E-04 |
| CPE | NM_001873 | -2.008 | 5,15E-04 |
| NR4A3 | NM_006981 | -2.008 | 1,43E-03 |
| IER3IP1 | NM_016097 | -2.011 | 9,92E-04 |
| RPL22 | NM_000983 | -2.011 | 1,15E-03 |
| SNAI2 | NM_003068 | -2.011 | 1,99E-03 |
| TXNDC1 | NM_030755 | -2.011 | 2,40E-03 |
| FHL2 | NM_001450 | -2.014 | 1,07E-03 |
| PSMA2 | NM_002787 | -2.016 | 7,10E-04 |
| KHDRBS1 | NM_006559 | -2.019 | 5,61E-03 |
| MEX3C | NM_016626 | -2.020 | 1,21E-03 |
| VCL | NM_003373 | -2.020 | 6,52E-04 |
| EIF4G2 | NM_001418 | -2.023 | 1,31E-03 |
| RCN1 | NM_002901 | -2.023 | 9,54E-04 |
| ARPP-19 | NM_006628 | -2.024 | 9,24E-04 |
| SERTAD2 | NM_014755 | -2.030 | 7,10E-04 |
| SMURF1 | NM_020429 | -2.030 | 1,00E-03 |
| SYNGR1 | NM_145738 | -2.030 | 3,89E-04 |
| CRK | NM_005206 | -2.031 | 4,13E-04 |
| HIST1H3H | NM_003536 | -2.041 | 3,03E-04 |
| GRSF1 | NM_002092 | -2.042 | 5,15E-04 |
| CALM1 | NM_006888 | -2.043 | 3,06E-03 |
| ERH | NM_004450 | -2.043 | 3,03E-04 |
| NUDCD2 | NM_145266 | -2.044 | 1,15E-03 |
| STAU1 | NM_004602 | -2.044 | 2,37E-04 |
| S100A13 | NM_005979 | -2.046 | 4,86E-04 |
| HSPD1 | NM_002156 | -2.048 | 2,55E-03 |
| CHSY1 | NM_014918 | -2.049 | 4,12E-03 |
| GPNMB | NM_002510 | -2.059 | 1,27E-03 |
| SFRS1 | NM_006924 | -2.059 | 6,06E-04 |
| CYBRD1 | NM_024843 | -2.065 | 1,84E-03 |
| GMPR | NM_006877 | -2.065 | 4,67E-04 |
| UBA2 | NM_005499 | -2.065 | 2,74E-03 |
| KYNU | NM_003937 | -2.067 | 3,22E-03 |
| NIPA2 | NM_030922 | -2.067 | 7,23E-04 |
| NPEPPS | NM_006310 | -2.067 | 3,04E-03 |
| OLFML2A | NM_182487 | -2.070 | 4,01E-03 |
| DNAJB6 | NM_005494 | -2.072 | 6,70E-04 |
| ABL1 | NM_005157 | -2.074 | 2,63E-04 |
| SEMA4C | NM_017789 | -2.077 | 9,51E-04 |
| TFF3 | NM_003226 | -2.077 | 3,31E-04 |
| TACC2 | NM_006997 | -2.079 | 7,82E-04 |
| GHRHR | NM_000823 | -2.080 | 1,62E-03 |
| LRPAP1 | NM_002337 | -2.087 | 1,57E-03 |
| MCL1 | NM_021960 | -2.087 | 2,83E-04 |
| H3F3A | NM_002107 | -2.093 | 2,54E-04 |
| ASAP2 | NM_003887 | -2.097 | 9,22E-04 |
| PFKP | NM_002627 | -2.102 | 2,17E-03 |
| TXNL1 | NM_004786 | -2.102 | 5,20E-04 |
| CCDC50 | NM_174908 | -2.106 | 8,54E-04 |
| PMPCB | NM_004279 | -2.106 | 8,21E-03 |
| IMPA1 | NM_005536 | -2.109 | 1,16E-03 |
| SRGAP2 | AB007925 | -2.110 | 5,90E-04 |
| KLHDC2 | NM_014315 | -2.115 | 7,00E-03 |
| ZRANB2 | NM_005455 | -2.117 | 2,54E-04 |
| CCT2 | NM_006431 | -2.120 | 6,17E-04 |
| H3F3B | NM_005324 | -2.120 | 1,57E-03 |
| MT1A | NM_005946 | -2.120 | 5,49E-03 |
| CORO1C | NM_014325 | -2.125 | 1,06E-03 |
| COG5 | NM_006348 | -2.126 | 5,94E-04 |
| PMEPA1 | NM_020182 | -2.132 | 1,84E-03 |
| YWHAH | NM_003405 | -2.135 | 1,90E-03 |
| JUND | NM_005354 | -2.137 | 2,16E-04 |
| CAMLG | NM_001745 | -2.139 | 6,46E-04 |
| MDFIC | AF054589 | -2.139 | 2,33E-03 |
| MRFAP1 | NM_033296 | -2.140 | 1,22E-03 |
| ZNF451 | NM_015555 | -2.142 | 2,36E-04 |
| NT5E | NM_002526 | -2.144 | 6,48E-04 |
| CAV1 | NM_001753 | -2.146 | 5,37E-04 |
| CNOT6 | NM_015455 | -2.146 | 2,92E-04 |
| ZMYND8 | NM_012408 | -2.146 | 3,02E-04 |
| IER3 | NM_003897 | -2.150 | 5,37E-04 |
| ST6GAL1 | NM_003032 | -2.150 | 5,76E-04 |
| RBBP7 | NM_002893 | -2.151 | 2,74E-04 |
| BDH2 | NM_020139 | -2.152 | 3,55E-04 |
| UGDH | NM_003359 | -2.152 | 1,09E-03 |
| CA14 | NM_012113 | -2.155 | 5,94E-04 |
| GINS2 | NM_016095 | -2.159 | 4,77E-04 |
| PTPN12 | NM_002835 | -2.159 | 6,37E-04 |
| CD164 | NM_006016 | -2.161 | 4,83E-04 |
| SIX1 | NM_005982 | -2.162 | 7,57E-04 |
| CALM2 | NM_001743 | -2.163 | 1,69E-03 |
| SUGT1 | NM_006704 | -2.164 | 1,03E-03 |
| CUL4A | NM_003589 | -2.165 | 2,97E-04 |
| RARS | NM_002887 | -2.170 | 7,06E-04 |
| UBE2K | NM_005339 | -2.171 | 3,72E-04 |
| KCTD20 | NM_173562 | -2.172 | 4,34E-04 |
| TIPRL | NM_152902 | -2.175 | 4,73E-03 |
| SHOX | NM_000451 | -2.177 | 2,78E-04 |
| FDPS | NM_002004 | -2.182 | 7,35E-04 |
| CCT5 | NM_012073 | -2.184 | 1,89E-03 |
| MED6 | NM_005466 | -2.189 | 2,97E-03 |
| GLO1 | NM_006708 | -2.191 | 2,10E-03 |
| FRMD6 | NM_152330 | -2.195 | 3,52E-04 |
| DHX15 | NM_001358 | -2.204 | 1,47E-03 |
| NCOA4 | NM_005437 | -2.206 | 1,16E-03 |
| PUM1 | NM_014676 | -2.206 | 4,67E-03 |
| PON2 | NM_000305 | -2.207 | 1,40E-03 |
| LAP3 | NM_015907 | -2.209 | 6,69E-04 |
| PRPS2 | NM_002765 | -2.209 | 5,25E-04 |
| PAPSS1 | NM_005443 | -2.210 | 1,55E-03 |
| JAG1 | NM_000214 | -2.211 | 5,75E-04 |
| TBCA | NM_004607 | -2.211 | 7,25E-04 |
| WDFY1 | NM_020830 | -2.212 | 2,23E-03 |
| EIF3H | NM_003756 | -2.213 | 1,37E-03 |
| MLLT10 | AF060929 | -2.215 | 2,36E-04 |
| REXO2 | NM_015523 | -2.217 | 4,28E-04 |
| LHFPL2 | NM_005779 | -2.219 | 2,12E-03 |
| MOAP1 | NM_022151 | -2.220 | 2,48E-03 |
| LRRK1 | NM_024652 | -2.222 | 1,37E-03 |
| TASP1 | NM_017714 | -2.222 | 6,79E-04 |
| PTP4A2 | NM_080391 | -2.223 | 2,53E-04 |
| SDC2 | NM_002998 | -2.225 | 6,69E-04 |
| IER5 | NM_016545 | -2.234 | 7,95E-04 |
| SERINC1 | NM_020755 | -2.236 | 4,39E-04 |
| CAPN2 | NM_001748 | -2.239 | 6,21E-03 |
| LSM14A | NM_015578 | -2.240 | 6,80E-04 |
| SH2B3 | NM_005475 | -2.240 | 3,40E-04 |
| ASMT | NM_004043 | -2.245 | 7,23E-04 |
| HNRNPU | NM_004501 | -2.246 | 2,88E-04 |
| RNF145 | NM_144726 | -2.253 | 3,90E-04 |
| LSAMP | NM_002338 | -2.260 | 8,60E-04 |
| PTPRM | NM_002845 | -2.260 | 5,67E-04 |
| AEBP1 | NM_001129 | -2.261 | 7,64E-04 |
| PXN | NM_002859 | -2.263 | 4,02E-04 |
| TIMM17B | BC029446 | -2.265 | 3,58E-04 |
| CREG1 | NM_003851 | -2.278 | 1,71E-03 |
| RNF13 | NM_007282 | -2.280 | 7,64E-04 |
| CNBP | NM_003418 | -2.289 | 2,16E-04 |
| DPY19L1 | AB020684 | -2.289 | 4,08E-03 |
| CDKN1B | NM_004064 | -2.298 | 2,16E-04 |
| RRM2 | NM_001034 | -2.299 | 2,78E-04 |
| ADNP | NM_015339 | -2.301 | 2,88E-04 |
| LYPLA1 | NM_006330 | -2.311 | 5,25E-04 |
| FYCO1 | NM_024513 | -2.313 | 5,15E-04 |
| AVPI1 | NM_021732 | -2.317 | 9,54E-04 |
| ADM | NM_001124 | -2.325 | 7,64E-04 |
| SLC15A4 | NM_145648 | -2.329 | 6,79E-04 |
| BECN1 | NM_003766 | -2.330 | 2,88E-04 |
| NBN | NM_002485 | -2.333 | 1,97E-04 |
| XPOT | NM_007235 | -2.334 | 2,38E-04 |
| SMNDC1 | NM_005871 | -2.340 | 3,02E-04 |
| MIA | NM_006533 | -2.345 | 4,29E-04 |
| PLK2 | AK098163 | -2.345 | 2,22E-04 |
| DNAJA1 | NM_001539 | -2.347 | 8,47E-04 |
| HNRNPK | NM_031262 | -2.353 | 7,60E-04 |
| FADS1 | NM_013402 | -2.363 | 1,31E-03 |
| PDCD5 | NM_004708 | -2.364 | 3,98E-03 |
| PTGES3 | NM_006601 | -2.364 | 8,03E-04 |
| LPHN1 | NM_024679 | -2.365 | 1,16E-03 |
| PHF19 | XM_045308 | -2.366 | 2,07E-03 |
| FNTB | NM_002028 | -2.367 | 4,39E-04 |
| UBP1 | NM_014517 | -2.378 | 1,15E-04 |
| PRKCH | NM_006255 | -2.387 | 1,66E-04 |
| RAD23B | NM_002874 | -2.399 | 1,15E-04 |
| VCAN | NM_004385 | -2.400 | 3,23E-04 |
| ZCCHC24 | NM_153367 | -2.401 | 6,31E-04 |
| PTPN1 | NM_002827 | -2.402 | 1,35E-04 |
| RAP1B | NM_015646 | -2.403 | 6,76E-03 |
| RNASEH2C | NM_032193 | -2.404 | 1,33E-04 |
| CLNS1A | NM_001293 | -2.406 | 2,88E-04 |
| SMAD2 | NM_005901 | -2.415 | 1,91E-03 |
| MID1 | NM_000381 | -2.416 | 1,35E-04 |
| ANXA7 | NM_004034 | -2.418 | 1,05E-03 |
| LRRC31 | AK097338 | -2.422 | 1,48E-03 |
| CTDSPL | NM_005808 | -2.425 | 1,31E-03 |
| C10ORF46 | NM_153810 | -2.427 | 4,39E-04 |
| PSMA3 | NM_002788 | -2.427 | 6,10E-04 |
| OLFM1 | NM_006334 | -2.430 | 2,65E-04 |
| SCARB2 | NM_005506 | -2.432 | 6,08E-04 |
| HCFC2 | NM_013320 | -2.435 | 1,22E-04 |
| WDR26 | NM_025160 | -2.437 | 1,28E-03 |
| RPL36AL | NM_001001 | -2.441 | 4,57E-04 |
| IFI16 | NM_005531 | -2.442 | 3,22E-04 |
| TUBB6 | NM_032525 | -2.443 | 5,93E-04 |
| CHMP1B | NM_020412 | -2.444 | 6,87E-04 |
| EIF4E | NM_001968 | -2.444 | 1,25E-03 |
| CNIH | NM_005776 | -2.448 | 2,04E-04 |
| HIAT1 | NM_033055 | -2.453 | 8,97E-04 |
| HMGCR | NM_000859 | -2.457 | 3,23E-04 |
| CCNG1 | NM_004060 | -2.465 | 3,52E-04 |
| C3 | NM_000064 | -2.466 | 3,31E-04 |
| MOBKL3 | NM_015387 | -2.466 | 5,40E-04 |
| USP14 | NM_005151 | -2.469 | 6,17E-04 |
| NOL7 | NM_016167 | -2.477 | 9,89E-04 |
| COL4A1 | NM_001845 | -2.483 | 5,59E-03 |
| FXYD3 | NM_005971 | -2.491 | 9,51E-04 |
| ERO1L | NM_014584 | -2.495 | 6,46E-04 |
| RPL15 | NM_002948 | -2.513 | 2,53E-04 |
| HEXIM1 | NM_006460 | -2.518 | 6,48E-04 |
| VBP1 | NM_003372 | -2.522 | 5,12E-04 |
| FLJ10357 | NM_018071 | -2.523 | 1,27E-03 |
| G3BP1 | NM_005754 | -2.526 | 1,93E-03 |
| ARL8B | NM_018184 | -2.527 | 1,24E-03 |
| CDC25B | NM_004358 | -2.530 | 1,37E-03 |
| XRCC5 | NM_021141 | -2.532 | 5,19E-04 |
| SNRPD1 | NM_006938 | -2.536 | 2,78E-03 |
| PNO1 | NM_020143 | -2.537 | 5,94E-04 |
| RCN2 | NM_002902 | -2.544 | 2,29E-04 |
| DPP4 | NM_001935 | -2.551 | 2,74E-04 |
| FKBP3 | NM_002013 | -2.557 | 3,02E-03 |
| RAB1A | NM_004161 | -2.567 | 1,43E-03 |
| IPO5 | NM_002271 | -2.570 | 1,16E-03 |
| HMGN1 | NM_004965 | -2.575 | 1,04E-04 |
| MGMT | NM_002412 | -2.580 | 4,06E-04 |
| GTPBP4 | NM_012341 | -2.589 | 3,72E-04 |
| ATP6AP2 | NM_005765 | -2.592 | 3,54E-04 |
| KLF5 | NM_001730 | -2.593 | 3,31E-04 |
| CPN1 | NM_001308 | -2.595 | 2,04E-04 |
| TNFRSF19 | NM_148957 | -2.596 | 3,43E-04 |
| TRIM44 | NM_017583 | -2.597 | 2,35E-04 |
| KIAA0649 | NM_014811 | -2.614 | 2,80E-04 |
| RIPK5 | XM_290898 | -2.619 | 2,67E-04 |
| SERBP1 | NM_015640 | -2.619 | 2,16E-04 |
| CAMSAP1 | AL834528 | -2.630 | 6,65E-04 |
| MYO10 | NM_012334 | -2.630 | 4,42E-03 |
| USP13 | NM_003940 | -2.630 | 2,85E-03 |
| DOT1L | NM_032482 | -2.631 | 1,47E-04 |
| NAT13 | NM_025146 | -2.634 | 1,70E-03 |
| TYMS | NM_001071 | -2.642 | 1,93E-03 |
| PGD | NM_002631 | -2.644 | 1,15E-04 |
| CYCS | NM_018947 | -2.645 | 1,33E-04 |
| UTP14A | NM_006649 | -2.647 | 2,99E-04 |
| VPS35 | NM_018206 | -2.651 | 5,57E-04 |
| SH3BP5 | NM_004844 | -2.655 | 8,55E-04 |
| TIMP3 | NM_000362 | -2.660 | 1,11E-04 |
| ADSS | NM_001126 | -2.682 | 9,24E-04 |
| OAT | NM_000274 | -2.705 | 4,29E-04 |
| RASIP1 | NM_017805 | -2.709 | 3,44E-04 |
| FOXJ3 | NM_014947 | -2.716 | 3,85E-04 |
| NPC1 | NM_000271 | -2.716 | 4,42E-04 |
| MAP1LC3B | NM_022818 | -2.721 | 1,33E-04 |
| NPDC1 | NM_015392 | -2.721 | 1,88E-04 |
| PTP4A1 | NM_003463 | -2.723 | 3,48E-03 |
| CTNNB1 | NM_001904 | -2.730 | 8,84E-05 |
| SAMD4A | NM_015589 | -2.730 | 4,26E-04 |
| MAGEC1 | NM_005462 | -2.734 | 6,28E-04 |
| PSMC1 | NM_002802 | -2.738 | 2,69E-04 |
| FAM101B | BC014203 | -2.741 | 8,14E-04 |
| TMEM22 | NM_025246 | -2.749 | 8,71E-03 |
| NSUN2 | NM_017755 | -2.751 | 3,72E-04 |
| APOLD1 | NM_030817 | -2.769 | 2,05E-03 |
| EIF2S3 | NM_001415 | -2.770 | 1,88E-04 |
| C1ORF38 | NM_004848 | -2.772 | 2,88E-04 |
| MAF | NM_005360 | -2.779 | 2,54E-04 |
| VAPA | NM_003574 | -2.779 | 1,18E-04 |
| HMGB1 | NM_002128 | -2.780 | 6,92E-04 |
| CNN3 | NM_001839 | -2.783 | 1,14E-03 |
| NPM1 | NM_002520 | -2.793 | 1,33E-04 |
| IGF2BP2 | NM_006548 | -2.794 | 1,39E-03 |
| CDYL | NM_004824 | -2.798 | 2,53E-04 |
| PPP1CC | NM_002710 | -2.809 | 2,19E-04 |
| RAC1 | NM_006908 | -2.815 | 2,51E-04 |
| FASTKD2 | NM_014929 | -2.822 | 1,15E-04 |
| IGFBP7 | NM_001553 | -2.825 | 1,56E-04 |
| MMP8 | NM_002424 | -2.832 | 1,93E-03 |
| TOR1AIP1 | NM_015602 | -2.836 | 1,85E-04 |
| TMEM2 | NM_013390 | -2.841 | 1,29E-03 |
| C11ORF58 | NM_014267 | -2.844 | 1,70E-03 |
| IL1RAP | NM_002182 | -2.852 | 1,33E-04 |
| CARD16 | NM_052889 | -2.855 | 2,60E-04 |
| LGALS3 | AF266280 | -2.869 | 4,39E-04 |
| GPR56 | NM_005682 | -2.872 | 4,12E-04 |
| KBTBD4 | NM_016506 | -2.875 | 6,27E-05 |
| TOMM20 | NM_014765 | -2.877 | 1,05E-03 |
| MFI2 | NM_033316 | -2.886 | 2,26E-04 |
| AP1S2 | NM_003916 | -2.888 | 6,49E-03 |
| SMN1 | NM_000344 | -2.890 | 2,74E-03 |
| SLC16A6 | NM_004694 | -2.893 | 9,29E-05 |
| SFPQ | NM_005066 | -2.895 | 5,84E-05 |
| HIRA | NM_003325 | -2.918 | 6,46E-04 |
| G3BP2 | NM_012297 | -2.919 | 3,40E-04 |
| JAM3 | NM_032801 | -2.927 | 3,98E-04 |
| PPP2CA | NM_002715 | -2.928 | 3,31E-04 |
| TMED10 | NM_006827 | -2.939 | 4,57E-04 |
| FAM20C | AL390147 | -2.952 | 3,55E-04 |
| HNRNPA2B1 | NM_002137 | -2.954 | 9,77E-05 |
| PABPC3 | NM_030979 | -2.956 | 3,72E-04 |
| MBP | BC030093 | -2.958 | 2,65E-04 |
| KLF6 | NM_001300 | -2.965 | 1,15E-04 |
| FOXO1 | NM_002015 | -2.978 | 2,63E-04 |
| AP2A1 | NM_014203 | -2.980 | 1,55E-03 |
| ASAH1 | NM_004315 | -2.983 | 2,29E-04 |
| YWHAB | NM_003404 | -2.986 | 2,31E-03 |
| FAM3C | NM_014888 | -2.993 | 1,93E-03 |
| RAB14 | NM_016322 | -2.993 | 9,25E-04 |
| FLJ11506 | NM_024666 | -2.999 | 2,99E-04 |
| HTRA1 | NM_002775 | -3.008 | 8,84E-05 |
| MRLC2 | NM_033546 | -3.016 | 3,31E-04 |
| CDV3 | NM_017548 | -3.019 | 2,53E-04 |
| PFDN4 | NM_002623 | -3.021 | 7,23E-04 |
| MAPK1IP1L | NM_144578 | -3.041 | 2,38E-04 |
| DSTN | NM_006870 | -3.062 | 4,31E-04 |
| PDZRN3 | XM_041363 | -3.062 | 2,16E-04 |
| SET | NM_003011 | -3.073 | 2,02E-04 |
| MAPRE1 | NM_012325 | -3.076 | 5,20E-03 |
| FAM108C1 | XM_051862 | -3.079 | 1,14E-04 |
| DNAJC6 | NM_014787 | -3.085 | 1,77E-04 |
| FAM46A | NM_017633 | -3.092 | 3,89E-04 |
| SDCBP | NM_005625 | -3.093 | 2,16E-04 |
| GNE | NM_005476 | -3.099 | 2,67E-04 |
| FN1 | NM_002026 | -3.105 | 6,64E-03 |
| OPLAH | AF217994 | -3.107 | 2,63E-04 |
| SRXN1 | NM_080725 | -3.110 | 1,15E-04 |
| TNC | NM_002160 | -3.129 | 5,94E-04 |
| MGC16385 | NM_032690 | -3.148 | 3,32E-03 |
| DLL3 | AK075302 | -3.151 | 1,39E-03 |
| ATG3 | NM_022488 | -3.172 | 2,54E-04 |
| HEXB | NM_000521 | -3.185 | 3,23E-04 |
| SRP19 | NM_003135 | -3.185 | 9,14E-05 |
| ACTN1 | NM_001102 | -3.189 | 3,44E-04 |
| SCCPDH | NM_016002 | -3.217 | 7,90E-04 |
| PAPOLA | NM_032632 | -3.236 | 3,32E-04 |
| SLC27A3 | NM_024330 | -3.242 | 2,16E-04 |
| MDK | NM_002391 | -3.246 | 1,88E-04 |
| DHRS7 | NM_016029 | -3.248 | 5,96E-03 |
| CCT6A | NM_001762 | -3.249 | 1,88E-04 |
| EIF5 | NM_001969 | -3.267 | 5,15E-04 |
| FGFR1 | NM_000604 | -3.269 | 5,55E-05 |
| RTKN2 | NM_145307 | -3.285 | 5,83E-05 |
| YWHAZ | NM_003406 | -3.325 | 5,55E-05 |
| RDH11 | NM_016026 | -3.340 | 2,69E-04 |
| FUBP3 | BC001325 | -3.388 | 1,32E-04 |
| ASS1 | NM_000050 | -3.391 | 5,00E-03 |
| PABPC1 | NM_002568 | -3.421 | 2,19E-03 |
| LOC552891 | NM_004125 | -3.507 | 5,84E-05 |
| HNRNPH2 | NM_019597 | -3.513 | 3,89E-04 |
| ZNF175 | NM_007147 | -3.520 | 1,88E-04 |
| UTP18 | NM_016001 | -3.533 | 2,16E-04 |
| COL4A2 | NM_001846 | -3.545 | 2,92E-03 |
| SARS2 | NM_017827 | -3.566 | 4,02E-04 |
| CDC16 | NM_003903 | -3.579 | 6,27E-05 |
| QPCT | NM_012413 | -3.586 | 5,21E-05 |
| NQO1 | NM_000903 | -3.603 | 6,89E-03 |
| TGIF1 | NM_170695 | -3.608 | 5,21E-05 |
| EIF3M | NM_006360 | -3.611 | 5,34E-04 |
| LEF1 | NM_016269 | -3.633 | 1,88E-04 |
| BBX | NM_020235 | -3.670 | 5,21E-05 |
| FCGRT | NM_004107 | -3.677 | 3,48E-03 |
| CASP1 | NM_033295 | -3.708 | 3,79E-03 |
| OLIG1 | BC026989 | -3.717 | 6,26E-04 |
| C14ORF166 | NM_016039 | -3.729 | 4,73E-04 |
| DYRK1A | NM_001396 | -3.744 | 2,97E-04 |
| CYTL1 | NM_018659 | -3.747 | 4,07E-04 |
| KPNB1 | NM_002265 | -3.760 | 2,65E-04 |
| PYCARD | NM_013258 | -3.784 | 5,98E-03 |
| FAIM3 | NM_005449 | -3.799 | 3,55E-04 |
| S100A10 | NM_002966 | -3.801 | 5,83E-05 |
| ARMCX2 | NM_014782 | -3.841 | 2,10E-04 |
| MTPN | NM_145808 | -3.883 | 2,26E-05 |
| UBE4A | NM_004788 | -3.924 | 9,77E-05 |
| S100B | NM_006272 | -3.967 | 1,60E-04 |
| BZW1 | NM_014670 | -4.054 | 1,93E-03 |
| PLA2G16 | NM_007069 | -4.059 | 4,29E-04 |
| GCSH | NM_004483 | -4.188 | 3,31E-04 |
| TDPX2 | X72297 | -4.286 | 4,10E-04 |
| RYK | NM_002958 | -4.345 | 5,51E-05 |
| BST2 | NM_004335 | -4.439 | 9,03E-05 |
| PDLIM3 | NM_014476 | -4.511 | 6,96E-05 |
| PHLDA2 | NM_003311 | -4.552 | 2,80E-04 |
| AHNAK | AK091153 | -4.574 | 3,22E-04 |
| CCND1 | NM_053056 | -4.663 | 1,15E-04 |
| DDX5 | NM_004396 | -4.700 | 5,84E-05 |
| PSD3 | NM_015310 | -4.702 | 6,03E-05 |
| COL22A1 | XM_291257 | -4.963 | 1,48E-05 |
| SEMA3B | NM_004636 | -4.982 | 2,64E-04 |
| SNRPB2 | NM_003092 | -5.052 | 1,83E-05 |
| TGFBI | NM_000358 | -5.139 | 2,31E-03 |
| CBX3 | NM_016587 | -5.364 | 3,32E-04 |
| SLC43A3 | NM_014096 | -5.796 | 3,73E-05 |
| MC1R | NM_002386 | -5.847 | 6,92E-05 |
| ZNF43 | AK097763 | -5.865 | 7,14E-05 |
| CCT8 | NM_006585 | -5.931 | 1,11E-04 |
| SEC24B | NM_006323 | -5.954 | 7,86E-05 |
| PRKAR1A | NM_002734 | -6.178 | 2,29E-04 |
| PRNP | NM_000311 | -6.193 | 5,21E-05 |
| APOD | NM_001647 | -6.843 | 5,21E-05 |
| PRDX1 | NM_002574 | -6.849 | 4,27E-05 |
| FABP5 | NM_001444 | -7.904 | 1,14E-05 |
| COL18A1 | NM_030582 | -8.355 | 1,14E-05 |
| MCAM | NM_006500 | -10.023 | 1,14E-05 |
| H1F0 | NM_005318 | -10.508 | 1,48E-05 |
| SYT6 | XM_086135 | -11.924 | 2,18E-05 |
| CXCR4 | NM_003467 | -12.243 | 1,00E-04 |
