# Supplementary material for: Gene expression profiles of human melanoma cells with different invasive potential reveal TSPAN8 as a novel mediator of invasion
Source: Br J Cancer. 2010 Nov 16;104(1):155–65. doi: 10.1038/sj.bjc.6605994 (PMC3039798; doi:10.1038/sj.bjc.6605994)
Supplement: Supplementary Table S3 [file 6605994x4.doc]

**Supplementary Table S3.** List of up-regulated genes and down-regulated functionally annotated to their implication in major diseases and disorders.

| **Diseases and Disorders** | | | | | | | | | | | | |
| --- | --- | --- | --- | --- | --- | --- | --- | --- | --- | --- | --- | --- |
| **Symbol** | **Description** | **GenBank** | **Fold Change** | **Cancer** | **Genetic Disorders** | **Inflammation** | **Metabolic Disease** | **Dermatological Disease** | | | | |
|  |  |  |  |  |  |  |  | **Melanoma** | **Dermatological Disorders** | **Basal-Cell Carcinoma** | **Hyperphasia** | **Skin Tumors** |
| **SERPINF1** | serpin peptidase inhibitor, clade F , member 1 | NM_002615 | **19,251** | X |  |  | X |  |  |  |  |  |
| **BCL11A** | B-cell CLL/lymphoma 11A (zinc finger protein) | NM_022893 | **17,664** | X | X |  |  |  |  |  |  |  |
| **RNLS** | renalase | NM_018363 | **16,745** | X | X |  |  |  |  |  |  |  |
| **MGST1** | microsomal glutathione S-transferase 1 | NM_020300 | **14,997** | X | X |  |  |  |  |  |  |  |
| **H2AFJ** | H2A histone family, member J | NM_177925 | **8,734** | X |  |  |  |  |  |  |  |  |
| **CAPG** | capping protein (actin filament), gelsolin-like | NM_001747 | **7,435** | X |  |  |  |  |  |  |  |  |
| **RELN** | reelin | NM_005045 | **6,782** | X | X |  |  |  |  |  |  |  |
| **SULT1A2** | sulfotransferase family, cytosolic, 1A, phenol-preferring, member 2 | NM_001054 | **6,08** | X | X |  |  |  |  |  |  |  |
| **PRDX2** | peroxiredoxin 2 | NM_005809 | **4,664** | X | X | X |  |  |  |  |  |  |
| **BAAT** | bile acid Coenzyme A: amino acid N-acyltransferase | NM_001701 | **4,393** |  | X |  |  |  |  |  |  |  |
| **IFITM2** | interferon induced transmembrane protein 2 (1-8D) | NM_006435 | **3,841** | X | X | X |  |  |  |  |  |  |
| **TSPAN8** | tetraspanin 8 | NM_004616 | **3,408** | X |  |  |  |  |  |  |  |  |
| **ID3** | inhibitor of DNA binding 3, dominant negative helix-loop-helix protein | NM_002167 | **3,337** | X | X | X |  |  |  |  |  |  |
| **PNOC** | prepronociceptin | NM_006228 | **3,072** |  | X |  |  |  |  |  |  |  |
| **FAM174B** | family with sequence similarity 174, member B | BC031970 | **2,843** | X |  |  |  |  |  |  |  |  |
| **FXYD5** | FXYD domain containing ion transport regulator 5 | NM_014164 | **2,821** | X |  |  |  |  |  |  |  |  |
| **SLC45A2** | solute carrier family 45, member 2 | NM_016180 | **2,672** |  | X |  |  | X | X |  |  |  |
| **ODC1** | ornithine decarboxylase 1 | NM_002539 | **2,651** | X | X | X |  | X | X | X | X | X |
| **GPI** | glucose phosphate isomerase | NM_000175 | **2,582** | X | X | X |  |  |  |  |  |  |
| **PCBD1** | pterin-4 alpha-carbinolamine dehydratase | NM_000281 | **2,553** |  | X |  | X |  |  |  |  |  |
| **STOX1** | storkhead box 1 | NM_152709 | **2,549** |  | X |  |  |  |  |  |  |  |
| **CD79B** | CD79b molecule, immunoglobulin-associated beta | NM_000626 | **2,432** | X | X | X |  | X | X |  |  |  |
| **CES1** | carboxylesterase 1 (monocyte/macrophage serine esterase 1) | NM_001266 | **2,43** | X |  |  |  |  |  |  |  |  |
| **FRZB** | frizzled-related protein | NM_001463 | **2,4** | X | X | X |  |  |  |  |  |  |
| **PUS7L** | pseudouridylate synthase 7 homolog (S. cerevisiae)-like | NM_031292 | **2,349** | X |  |  |  |  |  |  |  |  |
| **TYRP1** | tyrosinase-related protein 1 | NM_000550 | **2,32** |  | X |  |  | X | X |  |  |  |
| **HSD3B2** | hydroxy-delta-5-steroid dehydrogenase, 3 beta- and steroid delta-isomerase 2 | NM_000198 | **2,274** |  | X |  | X |  |  |  |  |  |
| **CNN2** | calponin 2 | NM_004368 | **2,242** | X |  |  |  |  |  |  |  |  |
| **HAND1** | heart and neural crest derivatives expressed 1 | NM_004821 | **2,235** | X | X |  |  |  |  |  |  |  |
| **KIAA1804** | mixed lineage kinase 4 | NM_032435 | **2,23** | X |  |  |  |  |  |  |  |  |
| **PPP2R1A** | protein phosphatase 2 (formerly 2A), regulatory subunit A, alpha isoform | NM_014225 | **2,189** | X |  |  |  |  |  |  |  |  |
| **CPT1C** | carnitine palmitoyltransferase 1C | NM_152359 | **2,179** |  | X |  |  |  |  |  |  |  |
| **ACTA2** | actin, alpha 2, smooth muscle, aorta | NM_001613 | **2,128** | X | X | X |  | X | X |  |  |  |
| **MRVI1** | murine retrovirus integration site 1 homolog | NM_006069 | **2,109** | X |  |  |  |  |  |  |  |  |
| **COX7B** | cytochrome c oxidase subunit VIIb | NM_001866 | **2,104** |  | X |  |  |  |  |  |  |  |
| **PDLIM4** | PDZ and LIM domain 4 | NM_003687 | **2,079** | X | X |  | X |  |  |  |  |  |
| **CYC1** | cytochrome c-1 | NM_001916 | **2,07** |  | X |  |  |  |  |  |  |  |
| **PLCD1** | phospholipase C, delta 1 | NM_006225 | **2,067** | X | X |  |  |  |  |  | X |  |
| **DNAJB1** | DnaJ (Hsp40) homolog, subfamily B, member 1 | NM_006145 | **2,066** | X | X |  |  |  |  |  |  |  |
| **ID1** | inhibitor of DNA binding 1, dominant negative helix-loop-helix protein | NM_002165 | **2,062** | X |  |  | X |  |  |  |  |  |
| **ICAM2** | intercellular adhesion molecule 2 | NM_000873 | **2,041** | X |  |  |  |  |  |  |  |  |
| **HPGD** | hydroxyprostaglandin dehydrogenase 15-(NAD) | NM_000860 | **2,035** | X | X | X |  |  |  |  |  |  |
| **UQCRQ** | ubiquinol-cytochrome c reductase, complex III subunit VII, 9.5kDa | NM_014402 | **2,03** |  | X |  | X |  |  |  |  |  |
| **ACO2** | aconitase 2, mitochondrial | NM_001098 | **2,025** |  |  | X |  |  |  |  |  |  |
| **RPS15** | ribosomal protein S15 | NM_001018 | **2,018** | X |  |  |  |  |  |  |  |  |
| **CD53** | CD53 molecule | NM_000560 | **2,011** | X |  |  |  |  |  |  |  |  |
| **KPNA2** | karyopherin alpha 2 (RAG cohort 1, importin alpha 1) | NM_002266 | **-2** | X | X |  |  |  |  |  |  |  |
| **MTHFD1** | methylenetetrahydrofolate dehydrogenase 1, formyltetrahydrofolate synthetase | NM_005956 | **-2,002** |  | X |  |  |  |  |  |  |  |
| **PSMB5** | proteasome (prosome, macropain) subunit, beta type, 5 | NM_002797 | **-2,004** | X | X | X | X | X |  |  |  | X |
| **CPE** | carboxypeptidase E | NM_001873 | **-2,008** |  | X |  |  |  |  |  |  |  |
| **NR4A3** | nuclear receptor subfamily 4, group A, member 3 | NM_006981 | **-2,008** |  | X | X | X |  |  |  |  |  |
| **RPL22** | ribosomal protein L22 | NM_000983 | **-2,011** | X |  |  |  |  |  |  |  |  |
| **SNAI2** | snail homolog 2 (Drosophila) | NM_003068 | **-2,011** | X | X |  |  | X | X |  |  |  |
| **FHL2** | four and a half LIM domains 2 | NM_001450 | **-2,014** | X | X |  |  |  |  |  |  |  |
| **PSMA2** | proteasome (prosome, macropain) subunit, alpha type, 2 | NM_002787 | **-2,016** | X |  |  |  |  |  |  |  |  |
| **MEX3C** | mex-3 homolog C (C. elegans) | NM_016626 | **-2,02** |  | X |  |  |  |  |  |  |  |
| **VCL** | vinculin | NM_003373 | **-2,02** | X | X |  |  |  |  |  |  |  |
| **EIF4G2** | eukaryotic translation initiation factor 4 gamma, 2 | NM_001418 | **-2,023** | X |  |  |  |  |  |  |  |  |
| **ARPP-19** | cyclic AMP phosphoprotein, 19 kD | NM_006628 | **-2,024** |  | X |  |  |  |  |  |  |  |
| **SERTAD2** | SERTA domain containing 2 | NM_014755 | **-2,03** | X |  |  |  |  |  |  |  |  |
| **SYNGR1** | synaptogyrin 1 | NM_145738 | **-2,03** | X | X |  |  |  |  |  |  |  |
| **CRK** | v-crk sarcoma virus CT10 oncogene homolog (avian) | NM_005206 | **-2,031** | X | X |  |  |  |  |  |  |  |
| **NUDCD2** | NudC domain containing 2 | NM_145266 | **-2,044** |  | X | X |  |  |  |  |  |  |
| **HSPD1** | heat shock 60kDa protein 1 (chaperonin) | NM_002156 | **-2,048** | X | X | X |  |  |  |  |  |  |
| **SFRS1** | splicing factor, arginine/serine-rich 1 | NM_006924 | **-2,059** | X |  |  |  |  |  |  |  |  |
| **NPEPPS** | aminopeptidase puromycin sensitive | NM_006310 | **-2,067** | X |  |  |  |  |  |  |  |  |
| **DNAJB6** | DnaJ (Hsp40) homolog, subfamily B, member 6 | NM_005494 | **-2,072** |  | X |  |  |  |  |  |  |  |
| **ABL1** | c-abl oncogene 1, receptor tyrosine kinase | NM_005157 | **-2,074** | X | X | X |  | X | X |  |  | X |
| **TFF3** | trefoil factor 3 (intestinal) | NM_003226 | **-2,077** | X | X | X |  |  |  |  |  |  |
| **GHRHR** | growth hormone releasing hormone receptor | NM_000823 | **-2,08** | X | X | X | X |  |  |  |  |  |
| **LRPAP1** | low density lipoprotein receptor-related protein associated protein 1 | NM_002337 | **-2,087** | X | X |  | X |  |  |  |  |  |
| **MCL1** | myeloid cell leukemia sequence 1 (BCL2-related) | NM_021960 | **-2,087** | X | X | X |  | X |  | X |  | X |
| **H3F3A** | H3 histone, family 3A | NM_002107 | **-2,093** |  | X | X |  | X | X |  |  |  |
| **PFKP** | phosphofructokinase, platelet | NM_002627 | **-2,102** | X | X |  |  |  |  |  |  |  |
| **CCDC50** | coiled-coil domain containing 50 | NM_174908 | **-2,106** |  | X |  |  |  |  |  |  |  |
| **IMPA1** | inositol(myo)-1(or 4)-monophosphatase 1 | NM_005536 | **-2,109** |  | X |  |  |  |  |  |  |  |
| **CCT2** | chaperonin containing TCP1, subunit 2 (beta) | NM_006431 | **-2,12** | X |  |  |  |  |  |  |  |  |
| **H3F3B** | H3 histone, family 3B (H3.3B) | NM_005324 | **-2,12** |  | X | X |  |  |  |  |  |  |
| **MT1A** | metallothionein 1A | NM_005946 | **-2,12** | X |  |  |  |  |  |  |  |  |
| **PMEPA1** | prostate transmembrane protein, androgen induced 1 | NM_020182 | **-2,132** | X |  |  |  |  |  |  |  |  |
| **YWHAH** | tyrosine 3monooxygenase/tryptophan 5monooxygenase activation protein, eta polypeptide | NM_003405 | **-2,135** |  | X |  |  |  |  |  |  |  |
| **JUND** | jun D proto-oncogene | NM_005354 | **-2,137** | X | X |  |  |  |  |  |  |  |
| **CAMLG** | calcium modulating ligand | NM_001745 | **-2,139** |  |  | X |  |  |  |  |  |  |
| **MRFAP1** | Mof4 family associated protein 1 | NM_033296 | **-2,14** |  | X | X |  |  |  |  |  |  |
| **CAV1** | caveolin 1, caveolae protein, 22kDa | NM_001753 | **-2,146** | X | X |  |  |  |  |  | X |  |
| **ZMYND8** | zinc finger, MYND-type containing 8 | NM_012408 | **-2,146** |  | X | X |  | X | X |  |  |  |
| **IER3** | immediate early response 3 | NM_003897 | **-2,15** | X | X | X |  |  |  |  |  |  |
| **ST6GAL1** | ST6 beta-galactosamide alpha-2,6-sialyltranferase 1 | NM_003032 | **-2,15** | X | X | X |  |  |  |  |  |  |
| **BDH2** | 3-hydroxybutyrate dehydrogenase, type 2 | NM_020139 | **-2,152** | X |  |  |  |  |  |  |  |  |
| **CA14** | carbonic anhydrase XIV | NM_012113 | **-2,155** |  | X | X | X |  |  |  |  |  |
| **GINS2** | GINS complex subunit 2 (Psf2 homolog) | NM_016095 | **-2,159** | X | X | X |  |  |  |  |  |  |
| **CD164** | CD164 molecule, sialomucin | NM_006016 | **-2,161** |  |  | X |  |  |  |  |  |  |
| **SIX1** | SIX homeobox 1 | NM_005982 | **-2,162** | X | X |  |  |  |  |  |  |  |
| **CUL4A** | cullin 4A | NM_003589 | **-2,165** | X |  |  |  |  |  |  |  |  |
| **SHOX** | short stature homeobox | NM_000451 | **-2,177** |  | X |  |  |  |  |  |  |  |
| **FDPS** | farnesyl diphosphate synthase | NM_002004 | **-2,182** | X | X |  | X |  |  |  |  |  |
| **CCT5** | chaperonin containing TCP1, subunit 5 (epsilon) | NM_012073 | **-2,184** | X | X |  |  |  |  |  |  |  |
| **GLO1** | glyoxalase I | NM_006708 | **-2,191** | X | X |  |  |  |  |  |  |  |
| **NCOA4** | nuclear receptor coactivator 4 | NM_005437 | **-2,206** | X |  |  |  |  |  |  |  |  |
| **JAG1** | jagged 1 (Alagille syndrome) | NM_000214 | **-2,211** | X | X | X |  |  |  |  |  |  |
| **MLLT10** | myeloid/lymphoid or mixed-lineage leukemia ; translocated to, 10 | AF060929 | **-2,215** | X | X |  |  |  |  |  |  |  |
| **MOAP1** | modulator of apoptosis 1 | NM_022151 | **-2,22** | X |  |  |  |  |  |  |  |  |
| **PTP4A2** | protein tyrosine phosphatase type IVA, member 2 | NM_080391 | **-2,223** | X |  |  |  |  |  |  |  |  |
| **SDC2** | syndecan 2 | NM_002998 | **-2,225** | X |  |  |  |  |  |  |  |  |
| **IER5** | immediate early response 5 | NM_016545 | **-2,234** |  | X |  |  |  |  |  |  |  |
| **SH2B3** | SH2B adaptor protein 3 | NM_005475 | **-2,24** | X |  | X |  |  |  |  |  |  |
| **HNRNPU** | heterogeneous nuclear ribonucleoprotein U (scaffold attachment factor A) | NM_004501 | **-2,246** |  | X |  |  |  |  |  |  |  |
| **PTPRM** | protein tyrosine phosphatase, receptor type, M | NM_002845 | **-2,26** | X |  |  |  |  |  |  |  |  |
| **AEBP1** | AE binding protein 1 | NM_001129 | **-2,261** |  | X |  |  |  |  |  |  |  |
| **PXN** | paxillin | NM_002859 | **-2,263** | X |  |  |  | X |  |  |  |  |
| **CREG1** | cellular repressor of E1A-stimulated genes 1 | NM_003851 | **-2,278** | X |  |  |  |  |  |  |  |  |
| **CNBP** | CCHC-type zinc finger, nucleic acid binding protein | NM_003418 | **-2,289** |  | X |  |  |  |  |  |  |  |
| **CDKN1B** | cyclin-dependent kinase inhibitor 1B (p27, Kip1) | NM_004064 | **-2,298** | X | X | X | X | X |  |  |  | X |
| **RRM2** | ribonucleotide reductase M2 polypeptide | NM_001034 | **-2,299** | X | X | X | X | X |  |  |  | X |
| **ADM** | adrenomedullin | NM_001124 | **-2,325** | X | X | X |  |  |  |  |  |  |
| **BECN1** | beclin 1, autophagy related | NM_003766 | **-2,33** | X |  | X |  |  |  |  |  |  |
| **NBN** | nibrin | NM_002485 | **-2,333** | X | X | X |  |  |  |  |  |  |
| **MIA** | melanoma inhibitory activity | NM_006533 | **-2,345** | X |  |  |  | X |  |  |  |  |
| **PLK2** | polo-like kinase 2 (Drosophila) | AK098163 | **-2,345** | X | X |  |  |  |  |  |  |  |
| **DNAJA1** | DnaJ (Hsp40) homolog, subfamily A, member 1 | NM_001539 | **-2,347** | X | X |  |  |  |  |  |  |  |
| **HNRNPK** | heterogeneous nuclear ribonucleoprotein K | NM_031262 | **-2,353** | X |  |  |  |  |  |  |  |  |
| **PTGES3** | prostaglandin E synthase 3 (cytosolic) | NM_006601 | **-2,364** | X | X |  |  |  |  |  |  |  |
| **LPHN1** | latrophilin 1 | NM_024679 | **-2,365** |  | X |  |  |  |  |  |  |  |
| **FNTB** | farnesyltransferase, CAAX box, beta | NM_002028 | **-2,367** | X | X |  |  | X |  |  |  | X |
| **PRKCH** | protein kinase C, eta | NM_006255 | **-2,387** | X | X |  |  | X | X |  |  |  |
| **RAD23B** | RAD23 homolog B (S. cerevisiae) | NM_002874 | **-2,399** | X |  |  |  |  |  |  |  |  |
| **VCAN** | versican | NM_004385 | **-2,4** | X | X |  |  |  |  |  |  |  |
| **PTPN1** | protein tyrosine phosphatase, non-receptor type 1 | NM_002827 | **-2,402** | X |  | X | X |  |  |  |  |  |
| **RNASEH2C** | ribonuclease H2, subunit C | NM_032193 | **-2,404** |  | X |  |  |  |  |  |  |  |
| **SMAD2** | SMAD family member 2 | NM_005901 | **-2,415** | X |  |  |  |  |  |  |  |  |
| **MID1** | midline 1 (Opitz/BBB syndrome) | NM_000381 | **-2,416** |  | X |  |  |  |  |  |  |  |
| **ANXA7** | annexin A7 | NM_004034 | **-2,418** | X |  |  |  |  |  |  |  |  |
| **CTDSPL** | carboxy-terminal domain, small phosphatase-like | NM_005808 | **-2,425** | X |  |  |  |  |  |  |  |  |
| **C10ORF46** | chromosome 10 open reading frame 46 | NM_153810 | **-2,427** | X |  |  |  |  |  |  |  |  |
| **OLFM1** | olfactomedin 1 | NM_006334 | **-2,43** | X | X |  |  |  |  |  |  |  |
| **SCARB2** | scavenger receptor class B, member 2 | NM_005506 | **-2,432** |  | X |  |  |  |  |  |  |  |
| **IFI16** | interferon, gamma-inducible protein 16 | NM_005531 | **-2,442** | X |  |  |  |  |  |  |  |  |
| **EIF4E** | eukaryotic translation initiation factor 4E | NM_001968 | **-2,444** | X |  | X |  |  |  |  |  |  |
| **CNIH** | cornichon homolog (Drosophila) | NM_005776 | **-2,448** |  |  | X |  |  |  |  |  |  |
| **HMGCR** | 3-hydroxy-3-methylglutaryl-Coenzyme A reductase | NM_000859 | **-2,457** | X | X | X | X | X |  |  |  |  |
| **CCNG1** | cyclin G1 | NM_004060 | **-2,465** | X |  |  | X |  |  |  |  |  |
| **C3** | complement component 3 | NM_000064 | **-2,466** | X | X | X | X |  |  |  |  |  |
| **NOL7** | nucleolar protein 7, 27kDa | NM_016167 | **-2,477** | X |  |  |  |  |  |  |  |  |
| **COL4A1** | collagen, type IV, alpha 1 | NM_001845 | **-2,483** | X | X | X | X | X | X |  |  |  |
| **RPL15** | ribosomal protein L15 | NM_002948 | **-2,513** |  | X |  |  |  |  |  |  |  |
| **CDC25B** | cell division cycle 25 homolog B (S. pombe) | NM_004358 | **-2,53** | X | X | X |  | X | X |  |  |  |
| **XRCC5** | X-ray repair complementing defective repair in Chinese hamster cells 5 | NM_021141 | **-2,532** | X |  | X |  |  |  |  |  |  |
| **DPP4** | dipeptidyl-peptidase 4 | NM_001935 | **-2,551** | X | X | X | X | X |  |  |  | X |
| **IPO5** | importin 5 | NM_002271 | **-2,57** |  | X |  |  |  |  |  |  |  |
| **HMGN1** | high-mobility group nucleosome binding domain 1 | NM_004965 | **-2,575** | X | X |  |  |  |  |  |  |  |
| **MGMT** | O-6-methylguanine-DNA methyltransferase | NM_002412 | **-2,58** | X |  | X |  | X |  |  |  | X |
| **GTPBP4** | GTP binding protein 4 | NM_012341 | **-2,589** | X |  |  |  |  |  |  |  |  |
| **ATP6AP2** | ATPase, H+ transporting, lysosomal accessory protein 2 | NM_005765 | **-2,592** |  | X |  |  |  |  |  |  |  |
| **KLF5** | Kruppel-like factor 5 (intestinal) | NM_001730 | **-2,593** | X |  |  |  |  |  |  |  |  |
| **CPN1** | carboxypeptidase N, polypeptide 1 | NM_001308 | **-2,595** |  | X | X |  |  |  |  |  |  |
| **SERBP1** | SERPINE1 mRNA binding protein 1 | NM_015640 | **-2,619** | X | X |  |  |  |  |  |  |  |
| **MYO10** | myosin X | NM_012334 | **-2,63** | X |  |  |  |  |  |  |  |  |
| **USP13** | ubiquitin specific peptidase 13 (isopeptidase T-3) | NM_003940 | **-2,63** |  | X |  |  |  |  |  |  |  |
| **TYMS** | thymidylate synthetase | NM_001071 | **-2,642** | X | X | X |  | X | X | X |  | X |
| **CYCS** | cytochrome c, somatic | NM_018947 | **-2,645** | X | X |  |  |  |  |  |  |  |
| **SH3BP5** | SH3-domain binding protein 5 (BTK-associated) | NM_004844 | **-2,655** |  |  | X |  |  |  |  |  |  |
| **TIMP3** | TIMP metallopeptidase inhibitor 3 | NM_000362 | **-2,66** | X | X | X | X |  |  |  |  |  |
| **ADSS** | adenylosuccinate synthase | NM_001126 | **-2,682** |  | X | X |  |  |  |  |  |  |
| **RASIP1** | Ras interacting protein 1 | NM_017805 | **-2,709** | X |  |  |  |  |  |  |  |  |
| **NPC1** | Niemann-Pick disease, type C1 | NM_000271 | **-2,716** | X | X | X | X |  |  |  |  |  |
| **NPDC1** | neural proliferation, differentiation and control, 1 | NM_015392 | **-2,721** | X | X |  |  |  |  |  |  |  |
| **PTP4A1** | protein tyrosine phosphatase type IVA, member 1 | NM_003463 | **-2,723** | X |  |  |  |  |  |  |  |  |
| **CTNNB1** | catenin (cadherin-associated protein), beta 1, 88kDa | NM_001904 | **-2,73** | X | X | X |  | X |  | X | X | X |
| **PSMC1** | proteasome (prosome, macropain) 26S subunit, ATPase, 1 | NM_002802 | **-2,738** |  | X |  |  |  |  |  |  |  |
| **FAM101B** | family with sequence similarity 101, member B | BC014203 | **-2,741** |  | X | X |  |  |  |  |  |  |
| **APOLD1** | apolipoprotein L domain containing 1 | NM_030817 | **-2,769** | X | X |  |  |  |  |  |  |  |
| **EIF2S3** | eukaryotic translation initiation factor 2, subunit 3 gamma, 52kDa | NM_001415 | **-2,77** | X | X |  |  |  |  |  |  |  |
| **MAF** | v-maf musculoaponeurotic fibrosarcoma oncogene homolog (avian) | NM_005360 | **-2,779** | X | X | X |  |  |  |  |  |  |
| **HMGB1** | high-mobility group box 1 | NM_002128 | **-2,78** | X | X | X |  | X | X |  |  |  |
| **NPM1** | nucleophosmin (nucleolar phosphoprotein B23, numatrin) | NM_002520 | **-2,793** | X | X | X |  |  |  |  |  |  |
| **IGF2BP2** | insulin-like growth factor 2 mRNA binding protein 2 | NM_006548 | **-2,794** |  | X |  | X |  |  |  |  |  |
| **PPP1CC** | protein phosphatase 1, catalytic subunit, gamma isoform | NM_002710 | **-2,809** | X |  |  |  |  |  |  |  |  |
| **RAC1** | ras-related C3 botulinum toxin substrate 1 | NM_006908 | **-2,815** | X | X | X |  | X | X |  |  |  |
| **IGFBP7** | insulin-like growth factor binding protein 7 | NM_001553 | **-2,825** | X | X | X | X |  |  |  |  |  |
| **MMP8** | matrix metallopeptidase 8 (neutrophil collagenase) | NM_002424 | **-2,832** |  |  | X |  |  |  |  |  |  |
| **TMEM2** | transmembrane protein 2 | NM_013390 | **-2,841** |  | X |  |  |  |  |  |  |  |
| **IL1RAP** | interleukin 1 receptor accessory protein | NM_002182 | **-2,852** | X |  | X |  |  |  |  |  |  |
| **CARD16** | caspase recruitment domain family, member 16 | NM_052889 | **-2,855** | X |  |  |  |  |  |  |  |  |
| **LGALS3** | lectin, galactoside-binding, soluble, 3 | AF266280 | **-2,869** | X | X | X | X | X |  |  |  |  |
| **GPR56** | G protein-coupled receptor 56 | NM_005682 | **-2,872** |  | X |  |  |  |  |  |  |  |
| **TOMM20** | translocase of outer mitochondrial membrane 20 homolog (yeast) | NM_014765 | **-2,877** |  | X |  |  |  |  |  |  |  |
| **MFI2** | antigen p97, identified by monoclonal antibodies 133.2 and 96.5 | NM_033316 | **-2,886** | X |  |  |  | X |  |  |  |  |
| **AP1S2** | adaptor-related protein complex 1, sigma 2 subunit | NM_003916 | **-2,888** |  | X |  |  |  |  |  |  |  |
| **SMN1** | survival of motor neuron 1, telomeric | NM_000344 | **-2,89** | X | X |  |  |  |  |  |  |  |
| **SFPQ** | splicing factor proline/glutamine-rich | NM_005066 | **-2,895** | X |  |  |  |  |  |  |  |  |
| **JAM3** | junctional adhesion molecule 3 | NM_032801 | **-2,927** | X |  | X |  |  |  |  |  |  |
| **PPP2CA** | protein phosphatase 2 (formerly 2A), catalytic subunit, alpha isoform | NM_002715 | **-2,928** | X |  |  |  |  |  |  |  |  |
| **TMED10** | transmembrane emp24-like trafficking protein 10 (yeast) | NM_006827 | **-2,939** |  | X |  |  |  |  |  |  |  |
| **MBP** | myelin basic protein | BC030093 | **-2,958** | X | X | X |  |  |  |  |  |  |
| **KLF6** | Kruppel-like factor 6 | NM_001300 | **-2,965** | X | X |  |  |  |  |  |  |  |
| **FOXO1** | forkhead box O1 | NM_002015 | **-2,978** | X | X | X | X | X | X |  |  |  |
| **ASAH1** | N-acylsphingosine amidohydrolase (acid ceramidase) 1 | NM_004315 | **-2,983** |  | X |  | X |  |  |  |  |  |
| **YWHAB** | tyrosine 3monooxygenase/tryptophan 5monooxygenase activation protein, beta polypeptide | NM_003404 | **-2,986** |  | X |  |  |  |  |  |  |  |
| **FAM3C** | family with sequence similarity 3, member C | NM_014888 | **-2,993** |  | X |  |  |  |  |  |  |  |
| **FLJ11506** | alpha- and gamma-adaptin-binding protein p34 | NM_024666 | **-2,999** | X |  |  |  |  |  |  |  |  |
| **HTRA1** | HtrA serine peptidase 1 | NM_002775 | **-3,008** | X | X |  |  |  |  |  |  |  |
| **DSTN** | destrin (actin depolymerizing factor) | NM_006870 | **-3,062** | X |  |  |  |  |  |  |  |  |
| **PDZRN3** | PDZ domain containing ring finger 3 | XM_041363 | **-3,062** | X |  |  |  |  |  |  |  |  |
| **SET** | SET nuclear oncogene | NM_003011 | **-3,073** | X | X |  |  |  |  |  |  |  |
| **MAPRE1** | microtubule-associated protein, RP/EB family, member 1 | NM_012325 | **-3,076** | X | X | X |  |  |  |  |  |  |
| **SDCBP** | syndecan binding protein (syntenin) | NM_005625 | **-3,093** | X |  |  |  | X |  |  |  |  |
| **GNE** | glucosamine (UDP-N-acetyl)-2-epimerase/N-acetylmannosamine kinase | NM_005476 | **-3,099** | X | X |  |  |  |  |  |  |  |
| **FN1** | fibronectin 1 | NM_002026 | **-3,105** | X | X | X | X | X | X |  |  |  |
| **TNC** | tenascin C | NM_002160 | **-3,129** | X | X | X |  |  |  |  |  |  |
| **DLL3** | delta-like 3 (Drosophila) | AK075302 | **-3,151** |  | X |  |  |  |  |  |  |  |
| **HEXB** | hexosaminidase B (beta polypeptide) | NM_000521 | **-3,185** |  | X |  | X |  |  |  |  |  |
| **MDK** | midkine (neurite growth-promoting factor 2) | NM_002391 | **-3,246** | X | X | X |  |  |  |  |  |  |
| **CCT6A** | chaperonin containing TCP1, subunit 6A (zeta 1) | NM_001762 | **-3,249** | X |  |  |  |  |  |  |  |  |
| **FGFR1** | fibroblast growth factor receptor 1 | NM_000604 | **-3,269** | X | X |  | X |  |  |  |  |  |
| **YWHAZ** | tyrosine 3monooxygenase/tryptophan 5monooxygenase activation protein, zeta polypeptide | NM_003406 | **-3,325** |  | X |  |  |  |  |  |  |  |
| **ASS1** | argininosuccinate synthetase 1 | NM_000050 | **-3,391** | X | X | X | X |  |  |  |  |  |
| **COL4A2** | collagen, type IV, alpha 2 | NM_001846 | **-3,545** | X | X | X |  | X | X |  |  |  |
| **QPCT** | glutaminyl-peptide cyclotransferase | NM_012413 | **-3,586** | X |  |  |  |  |  |  |  |  |
| **NQO1** | NAD(P)H dehydrogenase, quinone 1 | NM_000903 | **-3,603** | X | X | X |  |  |  |  |  |  |
| **TGIF1** | TGFB-induced factor homeobox 1 | NM_170695 | **-3,608** |  | X |  |  |  |  |  |  |  |
| **LEF1** | lymphoid enhancer-binding factor 1 | NM_016269 | **-3,633** | X |  |  |  |  |  |  |  | X |
| **FCGRT** | Fc fragment of IgG, receptor, transporter, alpha | NM_004107 | **-3,677** |  | X | X |  | X | X |  |  |  |
| **CASP1** | caspase 1, apoptosis-related cysteine peptidase | NM_033295 | **-3,708** | X | X | X |  | X | X |  |  |  |
| **DYRK1A** | dual-specificity tyrosine-(Y)-phosphorylation regulated kinase 1A | NM_001396 | **-3,744** | X |  |  |  |  |  |  |  |  |
| **PYCARD** | PYD and CARD domain containing | NM_013258 | **-3,784** | X |  |  |  |  |  |  |  |  |
| **S100A10** | S100 calcium binding protein A10 | NM_002966 | **-3,801** | X | X | X | X |  |  |  |  |  |
| **ARMCX2** | armadillo repeat containing, X-linked 2 | NM_014782 | **-3,841** |  | X |  |  |  |  |  |  |  |
| **UBE4A** | ubiquitination factor E4A (UFD2 homolog, yeast) | NM_004788 | **-3,924** | X |  |  |  |  |  |  |  |  |
| **S100B** | S100 calcium binding protein B | NM_006272 | **-3,967** | X | X |  |  |  |  |  |  |  |
| **BZW1** | basic leucine zipper and W2 domains 1 | NM_014670 | **-4,054** | X |  |  |  |  |  |  |  |  |
| **PLA2G16** | phospholipase A2, group XVI | NM_007069 | **-4,059** | X |  |  |  |  |  |  |  |  |
| **GCSH** | glycine cleavage system protein H (aminomethyl carrier) | NM_004483 | **-4,188** |  | X |  | X |  |  |  |  |  |
| **TDPX2** | thioredoxin-dependent peroxide reductase 2 | X72297 | **-4,286** | X |  |  |  |  |  |  |  |  |
| **RYK** | RYK receptor-like tyrosine kinase | NM_002958 | **-4,345** | X | X |  |  |  |  |  |  |  |
| **BST2** | bone marrow stromal cell antigen 2 | NM_004335 | **-4,439** | X | X | X |  |  |  |  |  |  |
| **PDLIM3** | PDZ and LIM domain 3 | NM_014476 | **-4,511** | X |  |  |  |  |  |  |  |  |
| **PHLDA2** | pleckstrin homology-like domain, family A, member 2 | NM_003311 | **-4,552** | X |  |  |  |  |  |  |  |  |
| **AHNAK** | AHNAK nucleoprotein | AK091153 | **-4,574** |  | X | X |  | X | X |  |  |  |
| **CCND1** | cyclin D1 | NM_053056 | **-4,663** | X | X | X | X | X |  |  | X | X |
| **DDX5** | DEAD (Asp-Glu-Ala-Asp) box polypeptide 5 | NM_004396 | **-4,7** | X |  |  |  |  |  |  |  |  |
| **COL22A1** | collagen, type XXII, alpha 1 | XM_291257 | **-4,963** |  |  |  |  | X | X |  |  |  |
| **SEMA3B** | sema domain, immunoglobulin domain, short basic domain, secreted, 3B | NM_004636 | **-4,982** | X |  |  |  |  |  |  |  |  |
| **TGFBI** | transforming growth factor, beta-induced, 68kDa | NM_000358 | **-5,139** |  | X |  |  |  |  |  |  |  |
| **MC1R** | melanocortin 1 receptor (alpha melanocyte stimulating hormone receptor) | NM_002386 | **-5,847** | X | X |  |  | X | X |  |  | X |
| **CCT8** | chaperonin containing TCP1, subunit 8 (theta) | NM_006585 | **-5,931** | X |  |  |  |  |  |  |  |  |
| **PRKAR1A** | protein kinase, cAMP-dependent, regulatory, type I, alpha | NM_002734 | **-6,178** | X | X |  | X |  |  |  |  |  |
| **PRNP** | prion protein | NM_000311 | **-6,193** | X | X | X |  |  |  |  |  |  |
| **APOD** | apolipoprotein D | NM_001647 | **-6,843** | X | X |  |  |  |  |  |  |  |
| **PRDX1** | peroxiredoxin 1 | NM_002574 | **-6,849** | X | X | X |  |  |  |  |  |  |
| **FABP5** | fatty acid binding protein 5 (psoriasis-associated) | NM_001444 | **-7,904** | X |  |  |  |  |  |  |  |  |
| **COL18A1** | collagen, type XVIII, alpha 1 | NM_030582 | **-8,355** | X | X |  |  | X | X |  |  |  |
| **MCAM** | melanoma cell adhesion molecule | NM_006500 | **-10,023** | X | X |  |  | X |  |  |  |  |
| **CXCR4** | chemokine (C-X-C motif) receptor 4 | NM_003467 | **-12,243** | X | X | X |  | X | X |  |  |  |
