# Supplementary material for: Gene expression profiles of human melanoma cells with different invasive potential reveal TSPAN8 as a novel mediator of invasion
Source: Br J Cancer. 2010 Nov 16;104(1):155–65. doi: 10.1038/sj.bjc.6605994 (PMC3039798; doi:10.1038/sj.bjc.6605994)
Supplement: Supplementary Table S4 [file 6605994x5.doc]

**Table S4**. List of up-regulated genes and down-regulated genes functionally annotated to their implication in molecular and cellular functions.

| **Supplementary Table S4** | | | | | | | | |
| --- | --- | --- | --- | --- | --- | --- | --- | --- |
| **Symbol** | **Entrez Gene Name** | **GenBank** | **FoldChange** | **Cell to Cell Signaling and Interaction** | **Cellular Assembly and Organisation** | **Cellular Growth and Cell Death** | **Cellular Movement and Invasion** | **DNA Replication, Recombination and Repair** |
| **SERPINF1** | serpin peptidase inhibitor, clade F (alpha-2 antiplasmin, pigment epithelium derived factor), member 1 | NM_002615 | **19.251** | X | X | X | X |  |
| **BCL11A** | B-cell CLL/lymphoma 11A (zinc finger protein) | NM_022893 | **17.664** | X | X |  | X |  |
| **RNLS** | renalase | NM_018363 | **16.745** | X |  |  |  |  |
| **MGST1** | microsomal glutathione S-transferase 1 | NM_020300 | **14.997** |  |  | X |  |  |
| **CAPG** | capping protein (actin filament), gelsolin-like | NM_001747 | **7.435** |  | X |  | X |  |
| **RELN** | reelin | NM_005045 | **6.782** | X | X |  | X |  |
| **PRDX2** | peroxiredoxin 2 | NM_005809 | **4.664** | X |  | X |  |  |
| **DENND2D** | DENN/MADD domain containing 2D | NM_024901 | **4.242** |  |  | X |  |  |
| **HERPUD1** | homocysteine-inducible, endoplasmic reticulum stress-inducible, ubiquitin-like domain member 1 | NM_014685 | **4.010** |  |  | X |  |  |
| **TSPAN8** | tetraspanin 8 | NM_004616 | **3.408** | X |  |  | X |  |
| **ID3** | inhibitor of DNA binding 3, dominant negative helix-loop-helix protein | NM_002167 | **3.337** | X |  | X | X | X |
| **PNOC** | prepronociceptin | NM_006228 | **3.072** |  |  |  | X |  |
| **FXYD5** | FXYD domain containing ion transport regulator 5 | NM_014164 | **2.821** | X |  |  | X |  |
| **ODC1** | ornithine decarboxylase 1 | NM_002539 | **2.651** |  |  | X | X |  |
| **GPI** | glucose phosphate isomerase | NM_000175 | **2.582** | X | X | X | X |  |
| **CD79B** | CD79b molecule, immunoglobulin-associated beta | NM_000626 | **2.432** |  |  | X |  |  |
| **CES1** | carboxylesterase 1 (monocyte/macrophage serine esterase 1) | NM_001266 | **2.430** |  |  | X |  |  |
| **FRZB** | frizzled-related protein | NM_001463 | **2.400** |  |  | X |  |  |
| **ABCC5** | ATP-binding cassette, sub-family C (CFTR/MRP), member 5 | NM_005688 | **2.366** |  |  | X |  |  |
| **PUS7L** | pseudouridylate synthase 7 homolog (S. cerevisiae)-like | NM_031292 | **2.349** |  |  | X |  | X |
| **TYRP1** | tyrosinase-related protein 1 | NM_000550 | **2.320** |  |  | X |  |  |
| **CNN2** | calponin 2 | NM_004368 | **2.242** |  |  | X | X |  |
| **HAND1** | heart and neural crest derivatives expressed 1 | NM_004821 | **2.24** |  |  |  | X |  |
| **PPP2R1A** | protein phosphatase 2 (formerly 2A), regulatory subunit A, alpha isoform | NM_014225 | **2.189** |  |  | X | X |  |
| **ACTA2** | actin, alpha 2, smooth muscle, aorta | NM_001613 | **2.128** | X |  |  | X |  |
| **PPIB** | peptidylprolyl isomerase B (cyclophilin B) | NM_000942 | **2.098** | X |  |  | X | X |
| **PDLIM4** | PDZ and LIM domain 4 | NM_003687 | **2.079** |  |  | X |  |  |
| **PLCD1** | phospholipase C, delta 1 | NM_006225 | **2.067** |  |  | X |  |  |
| **DNAJB1** | DnaJ (Hsp40) homolog, subfamily B, member 1 | NM_006145 | **2.066** |  |  | X |  |  |
| **ID1** | inhibitor of DNA binding 1, dominant negative helix-loop-helix protein | NM_002165 | **2.062** | X |  | X | X | X |
| **ICAM2** | intercellular adhesion molecule 2 | NM_000873 | **2.041** | X |  | X | X |  |
| **HPGD** | hydroxyprostaglandin dehydrogenase 15-(NAD) | NM_000860 | **2.035** |  |  | X |  |  |
| **CD53** | CD53 molecule | NM_000560 | **2.011** |  |  | X | X | X |
| **KPNA2** | karyopherin alpha 2 (RAG cohort 1, importin alpha 1) | NM_002266 | **-2.000** |  |  | X |  | X |
| **MTHFD1** | methylenetetrahydrofolate dehydrogenase 1 | NM_005956 | **-2.002** |  |  | X |  |  |
| **PSMB5** | proteasome (prosome, macropain) subunit, beta type, 5 | NM_002797 | **-2.004** |  |  | X |  |  |
| **NR4A3** | nuclear receptor subfamily 4, group A, member 3 | NM_006981 | **-2.008** |  |  | X |  | X |
| **SNAI2** | snail homolog 2 (Drosophila) | NM_003068 | **-2.011** |  |  | X | X |  |
| **TXNDC1** | thioredoxin domain containing 1 | NM_030755 | **-2.011** |  |  | X |  |  |
| **FHL2** | four and a half LIM domains 2 | NM_001450 | **-2.014** | X |  | X | X |  |
| **KHDRBS1** | KH domain containing, RNA binding, signal transduction associated 1 | NM_006559 | **-2.019** |  |  | X |  |  |
| **VCL** | vinculin | NM_003373 | **-2.020** | X | X | X | X |  |
| **EIF4G2** | eukaryotic translation initiation factor 4 gamma, 2 | NM_001418 | **-2.023** |  |  | X |  |  |
| **SERTAD2** | SERTA domain containing 2 | NM_014755 | **-2.030** |  |  | X |  |  |
| **SMURF1** | SMAD specific E3 ubiquitin protein ligase 1 | NM_020429 | **-2.030** | X |  |  |  |  |
| **SYNGR1** | synaptogyrin 1 | NM_145738 | **-2.030** |  | X |  |  |  |
| **CRK** | v-crk sarcoma virus CT10 oncogene homolog (avian) | NM_005206 | **-2.031** | X | X | X | X | X |
| **CALM1** | calmodulin 1 (phosphorylase kinase, delta) | NM_006888 | **-2.043** |  |  | X |  |  |
| **ERH** | enhancer of rudimentary homolog (Drosophila) | NM_004450 | **-2.043** |  |  | X |  |  |
| **STAU1** | staufen, RNA binding protein, homolog 1 (Drosophila) | NM_004602 | **-2.044** |  |  | X |  |  |
| **HSPD1** | heat shock 60kDa protein 1 (chaperonin) | NM_002156 | **-2.048** | X |  | X | X |  |
| **GPNMB** | glycoprotein (transmembrane) nmb | NM_002510 | **-2.059** | X |  | X |  |  |
| **SFRS1** | splicing factor, arginine/serine-rich 1 | NM_006924 | **-2.059** |  |  | X |  |  |
| **DNAJB6** | DnaJ (Hsp40) homolog, subfamily B, member 6 | NM_005494 | **-2.072** |  |  | X |  |  |
| **ABL1** | c-abl oncogene 1, receptor tyrosine kinase | NM_005157 | **-2.074** | X | X | X | X | X |
| **SEMA4C** | sema domain 4C | NM_017789 | **-2.077** |  |  | X | X |  |
| **TFF3** | trefoil factor 3 (intestinal) | NM_003226 | **-2.077** | X |  | X | X | X |
| **TACC2** | transforming, acidic coiled-coil containing protein 2 | NM_006997 | **-2.079** |  |  | X |  |  |
| **GHRHR** | growth hormone releasing hormone receptor | NM_000823 | **-2.080** |  |  | X |  |  |
| **LRPAP1** | low density lipoprotein receptor-related protein associated protein 1 | NM_002337 | **-2.087** | X |  | X | X |  |
| **MCL1** | myeloid cell leukemia sequence 1 (BCL2-related) | NM_021960 | **-2.087** |  |  | X | X | X |
| **ASAP2** | ArfGAP with SH3 domain, ankyrin repeat and PH domain 2 | NM_003887 | **-2.097** |  |  |  | X |  |
| **TXNL1** | thioredoxin-like 1 | NM_004786 | **-2.102** |  |  | X |  |  |
| **PMPCB** | peptidase (mitochondrial processing) beta | NM_004279 | **-2.11** |  |  |  | X |  |
| **CCT2** | chaperonin containing TCP1, subunit 2 (beta) | NM_006431 | **-2.120** |  |  | X |  |  |
| **MT1A** | metallothionein 1A | NM_005946 | **-2.120** |  |  | X |  |  |
| **PMEPA1** | prostate transmembrane protein, androgen induced 1 | NM_020182 | **-2.132** |  |  | X |  |  |
| **JUND** | jun D proto-oncogene | NM_005354 | **-2.137** |  |  | X | X |  |
| **NT5E** | 5'-nucleotidase, ecto (CD73) | NM_002526 | **-2.144** |  |  | X | X | X |
| **CAV1** | caveolin 1, caveolae protein, 22kDa | NM_001753 | **-2.146** | X |  | X | X | X |
| **IER3** | immediate early response 3 | NM_003897 | **-2.150** |  |  | X |  |  |
| **ST6GAL1** | ST6 beta-galactosamide alpha-2,6-sialyltranferase 1 | NM_003032 | **-2.150** | X |  | X | X |  |
| **RBBP7** | retinoblastoma binding protein 7 | NM_002893 | **-2.151** |  | X | X |  |  |
| **PTPN12** | protein tyrosine phosphatase, non-receptor type 12 | NM_002835 | **-2.159** |  |  |  | X |  |
| **CD164** | CD164 molecule, sialomucin | NM_006016 | **-2.161** | X |  | X | X |  |
| **SIX1** | SIX homeobox 1 | NM_005982 | **-2.162** |  |  | X | X |  |
| **SUGT1** | SGT1, suppressor of G2 allele of SKP1 (S. cerevisiae) | NM_006704 | **-2.164** |  |  | X |  |  |
| **CUL4A** | cullin 4A | NM_003589 | **-2.165** |  |  | X |  |  |
| **UBE2K** | ubiquitin-conjugating enzyme E2K (UBC1 homolog, yeast) | NM_005339 | **-2.171** |  |  | X |  |  |
| **SHOX** | short stature homeobox | NM_000451 | **-2.177** |  |  | X |  |  |
| **CDKN1C** | cyclin-dependent kinase inhibitor 1C (p57, Kip2) | NM_000076 | **-2.18** |  |  |  | X |  |
| **CCT5** | chaperonin containing TCP1, subunit 5 (epsilon) | NM_012073 | **-2.184** |  |  | X |  |  |
| **GLO1** | glyoxalase I | NM_006708 | **-2.191** |  |  | X |  |  |
| **NCOA4** | nuclear receptor coactivator 4 | NM_005437 | **-2.206** |  |  | X | X |  |
| **PON2** | paraoxonase 2 | NM_000305 | **-2.207** |  |  |  | X |  |
| **MLLT10** | myeloid/lymphoid or mixed-lineage leukemia; translocated to, 10 | AF060929 | **-2.21** |  |  |  | X |  |
| **WDFY1** | WD repeat and FYVE domain containing 1 | NM_020830 | **-2.21** |  |  |  | X |  |
| **JAG1** | jagged 1 (Alagille syndrome) | NM_000214 | **-2.211** | X |  | X | X |  |
| **MOAP1** | modulator of apoptosis 1 | NM_022151 | **-2.220** |  |  | X | X |  |
| **PTP4A2** | protein tyrosine phosphatase type IVA, member 2 | NM_080391 | **-2.223** |  |  | X | X |  |
| **SDC2** | syndecan 2 | NM_002998 | **-2.225** | X | X | X | X |  |
| **CAPN2** | calpain 2, (m/II) large subunit | NM_001748 | **-2.239** | X | X | X | X |  |
| **SH2B3** | SH2B adaptor protein 3 | NM_005475 | **-2.240** |  |  | X |  |  |
| **HNRNPU** | heterogeneous nuclear ribonucleoprotein U (scaffold attachment factor A) | NM_004501 | **-2.246** |  |  | X |  |  |
| **LSAMP** | limbic system-associated membrane protein | NM_002338 | **-2.26** |  |  |  | X |  |
| **PTPRM** | protein tyrosine phosphatase, receptor type, M | NM_002845 | **-2.260** | X | X | X |  |  |
| **AEBP1** | AE binding protein 1 | NM_001129 | **-2.261** |  |  | X |  |  |
| **PXN** | paxillin | NM_002859 | **-2.263** | X | X | X | X | X |
| **CREG1** | cellular repressor of E1A-stimulated genes 1 | NM_003851 | **-2.278** |  |  | X |  |  |
| **CNBP** | CCHC-type zinc finger, nucleic acid binding protein | NM_003418 | **-2.289** |  |  | X |  |  |
| **CDKN1B** | cyclin-dependent kinase inhibitor 1B (p27, Kip1) | NM_004064 | **-2.298** |  | X | X | X | X |
| **RRM2** | ribonucleotide reductase M2 polypeptide | NM_001034 | **-2.299** |  |  | X |  |  |
| **ADNP** | activity-dependent neuroprotector homeobox | NM_015339 | **-2.301** | X |  | X |  |  |
| **ADM** | adrenomedullin | NM_001124 | **-2.325** | X |  | X | X | X |
| **BECN1** | beclin 1, autophagy related | NM_003766 | **-2.330** |  |  | X | X |  |
| **NBN** | nibrin | NM_002485 | **-2.333** |  |  | X |  | X |
| **SMNDC1** | survival motor neuron domain containing 1 | NM_005871 | **-2.340** |  |  | X |  |  |
| **MIA** | melanoma inhibitory activity | NM_006533 | **-2.345** |  |  | X | X |  |
| **PLK2** | polo-like kinase 2 (Drosophila) | AK098163 | **-2.345** |  |  | X |  |  |
| **DNAJA1** | DnaJ (Hsp40) homolog, subfamily A, member 1 | NM_001539 | **-2.347** |  |  | X | X |  |
| **HNRNPK** | heterogeneous nuclear ribonucleoprotein K | NM_031262 | **-2.353** |  |  | X | X |  |
| **LPHN1** | latrophilin 1 | NM_024679 | **-2.36** |  |  |  | X |  |
| **FADS1** | fatty acid desaturase 1 | NM_013402 | **-2.363** |  |  | X |  |  |
| **PDCD5** | programmed cell death 5 | NM_004708 | **-2.364** |  |  | X |  |  |
| **PTGES3** | prostaglandin E synthase 3 (cytosolic) | NM_006601 | **-2.364** |  |  | X |  |  |
| **FNTB** | farnesyltransferase, CAAX box, beta | NM_002028 | **-2.367** |  |  | X |  |  |
| **PRKCH** | protein kinase C, eta | NM_006255 | **-2.387** |  |  | X |  |  |
| **RAD23B** | RAD23 homolog B (S. cerevisiae) | NM_002874 | **-2.399** |  |  | X |  |  |
| **VCAN** | versican | NM_004385 | **-2.400** | X | X | X | X |  |
| **PTPN1** | protein tyrosine phosphatase, non-receptor type 1 | NM_002827 | **-2.402** |  |  | X | X |  |
| **RAP1B** | RAP1B, member of RAS oncogene family | NM_015646 | **-2.403** |  | X | X |  | X |
| **SMAD2** | SMAD family member 2 | NM_005901 | **-2.415** | X | X | X | X |  |
| **ANXA7** | annexin A7 | NM_004034 | **-2.418** |  |  | X |  |  |
| **CTDSPL** | CTD small phosphatase-like | NM_005808 | **-2.425** |  |  | X |  |  |
| **C10ORF46** | chromosome 10 open reading frame 46 | NM_153810 | **-2.427** |  |  | X |  |  |
| **CASP1** | caspase 1, apoptosis-related cysteine peptidase (interleukin 1, beta, convertase) | NM_001223 | **-2.43** |  |  |  | X |  |
| **IFI16** | interferon, gamma-inducible protein 16 | NM_005531 | **-2.442** | X |  | X |  |  |
| **EIF4E** | eukaryotic translation initiation factor 4E | NM_001968 | **-2.444** |  |  | X |  |  |
| **HMGCR** | 3-hydroxy-3-methylglutaryl-Coenzyme A reductase | NM_000859 | **-2.457** |  |  | X | X |  |
| **CCNG1** | cyclin G1 | NM_004060 | **-2.465** |  |  | X |  |  |
| **C3** | complement component 3 | NM_000064 | **-2.466** | X |  | X | X |  |
| **COL4A1** | collagen, type IV, alpha 1 | NM_001845 | **-2.483** |  |  | X | X |  |
| **ERO1L** | ERO1-like (S. cerevisiae) | NM_014584 | **-2.495** |  |  | X |  |  |
| **CDC25B** | cell division cycle 25 homolog B (S. pombe) | NM_004358 | **-2.530** |  |  | X |  |  |
| **XRCC5** | X-ray repair complementing defective repair in Chinese hamster cells 5 | NM_021141 | **-2.532** |  |  | X | X | X |
| **DPP4** | dipeptidyl-peptidase 4 | NM_001935 | **-2.551** | X |  | X | X |  |
| **RAB1A** | RAB1A, member RAS oncogene family | NM_004161 | **-2.567** |  |  | X |  |  |
| **HMGN1** | high-mobility group nucleosome binding domain 1 | NM_004965 | **-2.575** |  |  | X |  |  |
| **MGMT** | O-6-methylguanine-DNA methyltransferase | NM_002412 | **-2.580** |  |  | X | X |  |
| **GTPBP4** | GTP binding protein 4 | NM_012341 | **-2.589** |  |  | X | X | X |
| **KLF5** | Kruppel-like factor 5 (intestinal) | NM_001730 | **-2.593** |  | X | X | X |  |
| **TNFRSF19** | tumor necrosis factor receptor superfamily, member 19 | NM_148957 | **-2.596** |  | X | X |  | X |
| **SERBP1** | SERPINE1 mRNA binding protein 1 | NM_015640 | **-2.619** | X |  | X |  |  |
| **MYO10** | myosin X | NM_012334 | **-2.630** |  | X |  | X |  |
| **TYMS** | thymidylate synthetase | NM_001071 | **-2.642** |  |  | X |  |  |
| **CYCS** | cytochrome c, somatic | NM_018947 | **-2.645** |  |  | X | X | X |
| **SH3BP5** | SH3-domain binding protein 5 (BTK-associated) | NM_004844 | **-2.655** |  |  | X |  |  |
| **TIMP3** | TIMP metallopeptidase inhibitor 3 | NM_000362 | **-2.660** | X |  | X | X |  |
| **NPC1** | Niemann-Pick disease, type C1 | NM_000271 | **-2.716** | X |  | X | X |  |
| **MAP1LC3B** | microtubule-associated protein 1 light chain 3 beta | NM_022818 | **-2.721** |  |  | X |  |  |
| **NPDC1** | neural proliferation, differentiation and control, 1 | NM_015392 | **-2.721** |  |  | X |  |  |
| **PTP4A1** | protein tyrosine phosphatase type IVA, member 1 | NM_003463 | **-2.723** |  |  | X | X |  |
| **CTNNB1** | catenin (cadherin-associated protein), beta 1, 88kDa | NM_001904 | **-2.730** | X | X | X | X |  |
| **PSMC1** | proteasome (prosome, macropain) 26S subunit, ATPase, 1 | NM_002802 | **-2.738** |  |  | X |  |  |
| **C1ORF38** | chromosome 1 open reading frame 38 | NM_004848 | **-2.772** | X |  |  |  |  |
| **MAF** | v-maf musculoaponeurotic fibrosarcoma oncogene homolog (avian) | NM_005360 | **-2.779** | X |  | X |  | X |
| **VAPA** | VAMP (vesicle-associated membrane protein)-associated protein A, 33kDa | NM_003574 | **-2.779** |  |  | X |  |  |
| **HMGB1** | high-mobility group box 1 | NM_002128 | **-2.780** | X | X | X | X |  |
| **NPM1** | nucleophosmin (nucleolar phosphoprotein B23, numatrin) | NM_002520 | **-2.793** |  |  | X | X |  |
| **PPP1CC** | protein phosphatase 1, catalytic subunit, gamma isoform | NM_002710 | **-2.809** |  |  | X |  |  |
| **RAC1** | ras-related C3 botulinum toxin substrate 1 (rho family, small GTP binding protein Rac1) | NM_006908 | **-2.815** | X | X | X | X | X |
| **IGFBP7** | insulin-like growth factor binding protein 7 | NM_001553 | **-2.825** | X |  | X |  |  |
| **MMP8** | matrix metallopeptidase 8 (neutrophil collagenase) | NM_002424 | **-2.832** |  |  | X | X |  |
| **LGALS3** | lectin, galactoside-binding, soluble, 3 | AF266280 | **-2.869** | X |  | X | X | X |
| **GPR56** | G protein-coupled receptor 56 | NM_005682 | **-2.872** | X |  | X | X |  |
| **MFI2** | antigen p97 (melanoma associated) identified by monoclonal antibodies 133.2 and 96.5 | NM_033316 | **-2.886** |  |  |  | X |  |
| **SMN1** | survival of motor neuron 1, telomeric | NM_000344 | **-2.890** |  | X | X |  |  |
| **HIRA** | HIR histone cell cycle regulation defective homolog A (S. cerevisiae) | NM_003325 | **-2.918** |  |  | X |  | X |
| **JAM3** | junctional adhesion molecule 3 | NM_032801 | **-2.927** | X |  |  | X |  |
| **PPP2CA** | protein phosphatase 2 (formerly 2A), catalytic subunit, alpha isoform | NM_002715 | **-2.928** |  |  | X |  |  |
| **HNRNPA2B1** | heterogeneous nuclear ribonucleoprotein A2/B1 | NM_002137 | **-2.954** |  |  | X |  |  |
| **MBP** | myelin basic protein | BC030093 | **-2.958** | X |  | X |  |  |
| **KLF6** | Kruppel-like factor 6 | NM_001300 | **-2.965** | X |  | X | X |  |
| **FOXO1** | forkhead box O1 | NM_002015 | **-2.978** |  |  | X | X |  |
| **ASAH1** | N-acylsphingosine amidohydrolase (acid ceramidase) 1 | NM_004315 | **-2.983** |  |  | X | X | X |
| **YWHAB** | tyrosine 3-monooxygenase/tryptophan 5-monooxygenase activation protein, beta polypeptide | NM_003404 | **-2.986** |  |  | X |  |  |
| **FLJ11506** | alpha- and gamma-adaptin-binding protein p34 | NM_024666 | **-2.999** |  |  | X |  | X |
| **HTRA1** | HtrA serine peptidase 1 | NM_002775 | **-3.008** |  |  | X |  |  |
| **MRLC2** | myosin regulatory light chain MRLC2 | NM_033546 | **-3.016** |  |  |  | X |  |
| **DSTN** | destrin (actin depolymerizing factor) | NM_006870 | **-3.062** |  |  | X | X |  |
| **SET** | SET nuclear oncogene | NM_003011 | **-3.073** |  |  | X |  | X |
| **MAPRE1** | microtubule-associated protein, RP/EB family, member 1 | NM_012325 | **-3.076** |  |  | X |  |  |
| **SDCBP** | syndecan binding protein (syntenin) | NM_005625 | **-3.093** |  |  |  | X |  |
| **GNE** | glucosamine (UDP-N-acetyl)-2-epimerase/N-acetylmannosamine kinase | NM_005476 | **-3.099** | X |  | X |  |  |
| **FN1** | fibronectin 1 | NM_002026 | **-3.105** | X | X | X | X | X |
| **TNC** | tenascin C | NM_002160 | **-3.129** | X | X | X | X | X |
| **HEXB** | hexosaminidase B (beta polypeptide) | NM_000521 | **-3.185** |  |  | X | X |  |
| **ACTN1** | actinin, alpha 1 | NM_001102 | **-3.189** |  |  | X |  |  |
| **MDK** | midkine (neurite growth-promoting factor 2) | NM_002391 | **-3.246** |  |  | X | X |  |
| **CCT6A** | chaperonin containing TCP1, subunit 6A (zeta 1) | NM_001762 | **-3.249** |  |  | X |  |  |
| **FGFR1** | fibroblast growth factor receptor 1 | NM_000604 | **-3.269** | X | X | X | X | X |
| **RTKN2** | rhotekin 2 | NM_145307 | **-3.285** |  |  | X |  |  |
| **YWHAZ** | tyrosine 3monooxygenase/tryptophan 5monooxygenase activation protein, zeta polypeptide | NM_003406 | **-3.325** |  | X | X | X |  |
| **COL4A2** | collagen, type IV, alpha 2 | NM_001846 | **-3.545** |  |  | X | X |  |
| **CDC16** | cell division cycle 16 homolog (S. cerevisiae) | NM_003903 | **-3.579** |  |  | X |  |  |
| **NQO1** | NAD(P)H dehydrogenase, quinone 1 | NM_000903 | **-3.603** |  |  | X |  |  |
| **TGIF1** | TGFB-induced factor homeobox 1 | NM_170695 | **-3.608** |  |  | X |  |  |
| **LEF1** | lymphoid enhancer-binding factor 1 | NM_016269 | **-3.633** |  |  | X | X |  |
| **CASP1** | caspase 1, apoptosis-related cysteine peptidase (interleukin 1, beta, convertase) | NM_033295 | **-3.708** |  |  | X |  |  |
| **C14ORF166** | chromosome 14 open reading frame 166 | NM_016039 | **-3.73** |  |  |  | X |  |
| **PYCARD** | PYD and CARD domain containing | NM_013258 | **-3.784** |  |  | X | X |  |
| **FAIM3** | Fas apoptotic inhibitory molecule 3 | NM_005449 | **-3.799** |  |  | X |  |  |
| **S100A10** | S100 calcium binding protein A10 | NM_002966 | **-3.801** | X |  | X | X |  |
| **MTPN** | myotrophin | NM_145808 | **-3.883** |  |  | X |  |  |
| **S100B** | S100 calcium binding protein B | NM_006272 | **-3.967** |  |  | X | X |  |
| **PLA2G16** | phospholipase A2, group XVI | NM_007069 | **-4.059** |  |  | X |  |  |
| **TDPX2** | thioredoxin-dependent peroxide reductase 2 | X72297 | **-4.286** |  |  | X |  |  |
| **RYK** | RYK receptor-like tyrosine kinase | NM_002958 | **-4.345** |  |  | X |  |  |
| **BST2** | bone marrow stromal cell antigen 2 | NM_004335 | **-4.439** |  |  | X |  |  |
| **PHLDA2** | pleckstrin homology-like domain, family A, member 2 | NM_003311 | **-4.552** |  |  | X |  |  |
| **CCND1** | cyclin D1 | NM_053056 | **-4.663** | X | X | X | X | X |
| **DDX5** | DEAD (Asp-Glu-Ala-Asp) box polypeptide 5 | NM_004396 | **-4.700** |  |  | X |  |  |
| **SEMA3B** | sema domain, immunoglobulin domain (Ig), short basic domain, secreted, (semaphorin) 3B | NM_004636 | **-4.982** | X |  | X | X |  |
| **TGFBI** | transforming growth factor, beta-induced, 68kDa | NM_000358 | **-5.139** | X |  | X | X |  |
| **MC1R** | melanocortin 1 receptor (alpha melanocyte stimulating hormone receptor) | NM_002386 | **-5.847** |  |  | X |  |  |
| **CCT8** | chaperonin containing TCP1, subunit 8 (theta) | NM_006585 | **-5.931** |  |  | X |  |  |
| **PRKAR1A** | protein kinase, cAMP-dependent, regulatory, type I, alpha (tissue specific extinguisher 1) | NM_002734 | **-6.178** | X |  | X | X |  |
| **PRNP** | prion protein | NM_000311 | **-6.193** | X | X | X | X | X |
| **PRDX1** | peroxiredoxin 1 | NM_002574 | **-6.849** |  |  | X |  |  |
| **FABP5** | fatty acid binding protein 5 (psoriasis-associated) | NM_001444 | **-7.904** |  |  | X | X |  |
| **COL18A1** | collagen, type XVIII, alpha 1 | NM_030582 | **-8.355** | X | X | X | X |  |
| **MCAM** | melanoma cell adhesion molecule | NM_006500 | **-10.023** | X |  | X | X |  |
| **CXCR4** | chemokine (C-X-C motif) receptor 4 | NM_003467 | **-12.243** | X | X | X | X |  |
