# Supplementary material for: Gene expression profiles of human melanoma cells with different invasive potential reveal TSPAN8 as a novel mediator of invasion
Source: Br J Cancer. 2010 Nov 16;104(1):155–65. doi: 10.1038/sj.bjc.6605994 (PMC3039798; doi:10.1038/sj.bjc.6605994)
Supplement: Supplementary Table S5 [file 6605994x6.doc]

**Table S5.** TSPAN8 staining of nevi and melanoma paraffin-embedded tissue sections.

| **Type of lesion** | **TSPAN8 positive/total** |
| --- | --- |
| Normal skin | 0/8 |
| Benign nevi (compound, congenital) | 8/16 |
| Malignant |  |
| RGP primary melanoma | 8/13 |
| VGP primary melanoma | 10/35 |
| Metastatic melanoma (lymph nodes) | 2/6 |

Normal skin, benign nevi, primary melanomas and lymph node metastases were incubated with anti-TSPAN8 antibody and the catalyzed amplification system, as described above in "Materials and Methods". Results are shown as number of positive lesions per number of tested lesions.
