# Supplementary material for: Gene expression profiles of human melanoma cells with different invasive potential reveal TSPAN8 as a novel mediator of invasion
Source: Br J Cancer. 2010 Nov 16;104(1):155–65. doi: 10.1038/sj.bjc.6605994 (PMC3039798; doi:10.1038/sj.bjc.6605994)
Supplement: Supplementary Information [file 6605994x7.doc]

**Supplemental Materials and Methods**

**Antibodies and reagents**

The following antibodies were used: mouse anti-RELN (E-5; Santa Cruz Biotechnology inc., Santa Cruz, CA); mouse anti-ID3 (2B11; Novus Biologicals, Littleton, CO); mouse anti-CAPG (1F1) and mouse polyclonal anti-BCL11A (A01) from Abnova Laboratories (Taipei, Taiwan); rabbit polyclonal anti-FXYD5 (Aviva Antibody, San Diego, CA) and goat polyclonal anti-C10orf59 (Imgenex, San Diego, CA). The mouse monoclonal antibodies directed against tetraspanins were TS9 (CD9), TS53 (CD53), TS63 (CD63), TS81 (CD81), TS82 (CD82), TS151 (CD151) and TS29 (Tspan8) (Azorsa *et al*., 1999; Le Naour *et al.*, 2006). Accutase was purchased from Sigma Chemical Co. (St. Louis, MO). The secondary antibodies were: goat IgG anti-mouse coupled either to peroxidase (Calbiochem, San Diego, CA) or to FITC (Beckman Coulter, Inc. Fullerton, CA); donkey anti-rabbit AF488 (Molecular Probes); goat anti-rabbit coupled peroxidase (Calbiochem); rabbit anti-goat IgG, F(ab’)2 fragment coupled to peroxidase (Santa Cruz Biotechnology, Santa cruz, CA) or FITC (Zymed Laboratories, South San Francisco, CA). Mouse, rabbit and goat IgG mAbs were all purchased from Pharmingen (Hamburg, Germany).

**Cell lines and cell culture**

The M4Be, M3Da, M1Do, M2Ge cell lines were established from metastases of four patients with malignant melanoma (Jacubovich and Doré, 1979). These cell lines did not express PNA-binding sites and are unable to give lung metastases after orthotopic injection into immunosuppressed newborn rats (unpublished data) and did not invade dermis in skin reconstructs (Béchetoille *et al*, 2000; Goidin *et al*, 2001). Six clones and variants, previously selected from the parental M4Be cell line, were choosen for their ability to give high (T1C3, TW12, T1P26) or low (IC8, T1C11) numbers of spontaneous metastases in the lungs of immunosuppressed newborn rats (Bailly and Doré, 1991; Bailly and Doré, 1992; Zebda *et al.,* 1994; Zebda *et al.,* 1995). Clones T1C3, TW12, T1C11, and IC8 were generated by limiting dilution cloning of M4Be cell lines whereas T1P26 variant was selected by *in* *vivo* serial passages of several cycles of lung metastases, as described previously (Bailly and Doré, 1991; Bailly and Doré, 1992). Melanoma cells from T1C3 and TW12 clones are able to invade dermis in skin reconstructs, in contrast to their parental M4Be cell line and the clone IC8 (Béchetoille et al, 2000). Clones and variants have a doubling time (30 hours ± 0.5) similar to that of the parent M4Be line. Their common origin is ascertained by their karyotype similar to that of the parental M4Be cell line (Bailly and Doré, 1992; Bailly *et al*, 1993).

Normal melanocytes obtained from three infant foreskins were cultured in serum-free MM-4 medium, as described elsewhere (Valéry *et al.,* 2001). The highly invasive human melanoma cell lines WM 793 and SK Mel28 were from the American Type Culture Collection (ATCC, Manassas, VA). Melanoma cells were routinely cultured as monolayers in either McCoy’s 5A medium (Gibco, Paisley, Scotland) or DMEM supplemented with 10% fetal calf serum, 100 IU/ml penicillin and 100 IU/ml streptomycin. LOVO colon carcinoma cell line, generously supplied by Dr. Lionel Rémy (INSERM U865, Lyon, France), was used as positive control for TSPAN8 expression. DAUDI, MeWo, HT29 and HeLa cell lines obtained from the ATCC, were used as positive control for the gene products of BCL11A, CAPG, FXYD5 and ID3, respectively. Cultures were regularly screened for mycoplasma contamination using the Hoechst 33258 fluorescence staining procedure.

**DNA microarray hybridization and data analysis**

Total RNA from IC8 and T1C3 clones was extracted using Tripure reagent (Roche, Germany). RNA quality was checked by 1% agarose gel electrophoresis and concentration was measured by absorbance at 260 nm. Extracted RNA was further amplified using the AminoAllyl MessageAmp II aRNA amplification kit (Ambion) following the manufacturer’s instructions. Hybridization and data analysis were performed as described earlier by us (Bonin *et al*., 2009). Briefly, for each hybridization, 1 μg of amplified RNA from IC8 and T1C3 cells was labeled by an indirect method using monofunctional NHS-ester Cy3 or Cy5 (GE Healthcare Life Sciences). Four independent dye-swap hybridizations (eight microarrays) were performed. The microarrays produced by the CEA (French atomic energy authority, Evry, France) contained a total of 26 068 long oligos (50mer) representing 21 000 human genes (Bonin *et al*., 2009). Slides were scanned on a Genepix 4000B microarray scanner (Axon Instruments, Molecular Devices Corp.). For each hybridized spot, the Cy3 and Cy5 fluorescence values were obtained by Genepix Pro 6.0 software (Molecular Devices Corp.) and saved as a results file. Spots or areas of the array with obvious blemishes were manually flagged and excluded from subsequent analysis. Results files were imported into Genespring GX 7.3.1 (Agilent) for further analysis. Two normalization steps, dye-swap data transformation and intensity-dependent Lowess were applied in order to eliminate dye-related bias in the two-color experiments. Statistical significance of expression ratios was calculated using the Student's t test. The list of differentially expressed genes was submitted to Ingenuity Pathway Analysis 5.5 (www.ingenuity.com) to analyze gene functions. The significance of functional enrichment was computed by a Fisher's exact test and represented by a range of P-values associated with specific functions. Differentially expressed genes were selected as described in the Results section. All the microarray data have been deposited into the GEO database at http://www.ncbi.nlm.nih.gov/projects/geo/ and are available under the accession number GSE15802.

**Semiquantitative RT-PCR**.

Total extracted RNA was reverse-transcribed using random hexamers and Superscript III reverse transcriptase (Invitrogen), following the manufacturer-recommended protocol. All RT reactions were performed on the MJ Research PTC-200 thermocycler (MJ research Inc.). For RELN, C10orf59, CAPG, H2AFJ and FXYD5, semi-quantitative PCR was carried out in a Peltier thermal cycler (MJ Research Inc.) using the following specific primer sets obtained from the sequences in EST databases: RELN, 5-TCCAGGCTCTCCTCTTACCA (forward) and 5’AGATTTGCTCCCCAGTCTCA3’(reverse); C10orf59, 5'CGCCTATTGAAGGAATGGTG3' (forward) and 5'AGGGGAGCTGTTTCTTT3' (reverse); CAPG, 5’ACACAGCCATTCCCCAGAGT3’ (forward) and 5’AGTCCCCCGAGAAGAAGACG3’ (reverse) ; H2AFJ 5'GGAGTACCTTACGGCGGAGA3' (forward) and 5’CCCTGAGCGATGGTCACTTT3’ (reverse) ; FXYD5, 5’TCTGGTCGCCTGTGTCTTCT3’(forward) and 5’GAGAGGTGGGCTGGAGTTCT3’. The PCR exponential phase was determined on 23-37 cycles to allow semi-quantitative comparisons of cDNAs developed from identical reactions with Taq DNA polymerase (Invitrogen). The PCR products were resolved on 2% agarose gels using ethidium bromide staining.

**Quantitative** **real-time RT-PCR**

Real-time PCR were performed on an Mx3000P real-time PCR system (Stratagene) using the RT-PCR products as templates. Brilliant SYBR Green QPCR Master Mix (Stratagene) was used in all qPCR reactions. The following primers were used: 5’-TTGCTTCTGATCCTGCTCCT-3’ and 5′-AGG GCC TGC AGG TTC ACA CCA C-3′ for TSPAN8, producing a 135 bp fragment ; 5’-AACCCCAGCACTTAAGCAAA-3’ and 5‘-CCAATGGGAAGTTCATCTGG-3’ for BCL11A producing a 150 bp fragment ; 5’-TGAGCTTGCTGGACGACATG-3’ and 5’-GATGACGCGCTGTAGGATTTC-3’ for ID3 producing a 150 bp fragment  and 5’-CGATGCGGCGGCGTTATT-3' and 5'-CCTGGTCTGTCTCATCCTCCC-3' for 18S producing a 150 bp fragment. A dissociation curve program was applied to all samples to check for specific Q-PCR products and avoid primer-dimmer or non-specific Q-PCR products. Because the expression levels of classical housekeeping genes were not similar between the two clones (Goidin et al., 2001), all transcript levels were normalized to that of 18S rRNA and quantified by using the ΔΔCt value method (Mx Pro software). Three biological replicates, resulting from three different RNA extractions were used for quantification analysis and three technical replicates were analyzed for each biological replicate. Fold differences were calculated using the mathematical model described by Pfaffl *et al.* (2001).

**Flow cytometry.**

Melanoma cells (1 x 10^5^) were washed in PBS containing 3% FCS and incubated for 20 min at 4°C with primary antibodies directed against FXYD5, TSPAN8, CD9, CD53, CD63, CD81, CD82 and CD151 at the appropriate concentration or with irrelevant isotype-matched Igs at the same concentration. Intracellular staining for CAPG, renalase, BCL11A, RELN and ID3 was carried out using the cytofix and cytoperm cell permeabilization kit (BD Biosciences, Le Pont de Claix, France) according to the manufacturer’s instructions. After incubation with fluorescein isothiocyanate (FITC)-conjugated F(ab)'2 fragments of goat anti-mouse antibody (Beckman Coulter), flow cytometry was conducted on FACS calibur using Cellquest software (BD Biosciences). The results are reported as mean fluorescence intensity of three independent experiments.

**Immunohistochemical analysis of normal skin and melanoma tissues**

Formalin-fixed and paraffin-embedded melanocytic lesions were obtained from the archives of the Department of Pathology of the Centre Léon Bérard (Lyon, France). Lesions comprised 16 benign nevi (8 compound and 8 congenital), 13 primary melanomas in RGP, 35 primary melanomas that had entered VGP and 6 metastatic melanomas to lymph nodes. The tumor thickness was under 0,75 mm in all the melanomas in RGP and over 1 mm in all the melanomas in VGP. Primary melanomas were considered to be in RGP if no dermal mitotic figures were present, dermal nests of melanocytes were smaller than the largest intra-epidermal nests and invasion did not reach the deepest part of the papillary dermis. By contrast, VGP is defined by the presence of melanoma cells expanding into the dermis and generating significant tumor nodules. Serial 4-µm paraffin-embedded tissue sections were screened for TSPAN8 protein expression by a biotin-streptavidin-amplified technique with an alkaline phosphatase kit (Dako-LSAB2- System, DAKO, Hamburg, Germany) using an automated immunostainer (Ventana, Tucson, AZ). Isotype control mouse IgG was used as negative control and bound antibody was detected with fast red, which gives rise to a red chromogen. Sections were counterstained with hematoxylin and finally mounted in aqueous mounting medium. Staining of eccrine glands in the dermis served as positive controls. Tissue sections were scored positive if any reactive areas were seen.

**Cell viability assay**

Graded numbers of melanoma cells transfected or not with TSPAN8–specific or scramble siRNAs were plated in culture medium in 96-wells plates and cultured for a further 2 days. Cell viability was determined by means of a colorimetric XTT viability assay (Sigma Chemical Co., St. Louis, MO) in accordance with the supplier's instructions. Absorbance at 450 nm was measured using a plate reader (Victor 3, Perkin Elmer, Shelton, CT) and cell viability was expressed as a mean of four replicate wells from 3 independent experiments.

**Cell proliferation assay**

Varying numbers of post-siRNA transfection cells were plated in 96-well plates and cultured for 24 hr. BrdU was or was not added to the medium, and 6 hr later, the amount of incorporated BrdU was determined by the Cell Proliferation ELISA BrdU colorimetric assay (Millipore, Mannheim, Germany) according to the manufacturer’s instructions. This non-radioactive system detects the amount of BrdU within chromosomal DNA by peroxidase-labeled anti-BrdU antibody-colored 3,3',5,5'-tetramethylbenzidine. Cultured cells without BrdU addition were used as controls for non-specific binding. Optic absorption at 450 nm was measured to detect BrdU, using a plate reader (Victor 3, Perkin Elmer, Shelton, CT, USA). Three independent experiments were performed and the extent of proliferation was expressed as OD mean values in the presence of BrdU - OD mean values without BrdU ± SD of triplicate wells.

**Migration Assay**

Migration of melanoma cells was assessed using a mechanical scratch wound assay. Briefly, monolayers of confluent cells in six-well plates were scraped using a sterile micropipette tip. The wells were rinsed twice with PBS to remove detached cells and fresh complete medium was replaced. Migration of cells was observed with an inverted microscope and photographed. For each siRNA transfection, phase-contrast images of three to four different cleared zones per well were captured immediately after wounding (0 hr) and periodically recorded at the same location over the next 48 hr. The cell-free area of each monolayer was distinguished from the surrounding intact cells by applying an automatic, software-defined threshold to each image, with pseudo-color applied to the resulting areas by computer image analysis (NIH Image J analysis software). Two independent experiments were performed and the relative re-colonization of the initially cell-free scratch was quantified and calculated as follows: (area at 0 hr - area at each time point)/area at 0 hr X 100.

**Three-dimensional spheroid invasion assay.**

Spheroid cell culture was performed using the hanging drop method, as described previously (Smalley *et al.*, 2006). Briefly, melanoma cells were detached from the flask using accutase, resuspended in Mac Coy’5A containing 10% FBS and centrifuged. Hanging drop culture was used, where 2 x 10^6^ melanoma cells/mL were seeded (1 µl droplets) into a thick layer of undiluted matrigel (300 µL/well; Becton Dickinson) partially polymerized in an 8-well chamber slide (Lab Tek, Nalge Nunc International, Rochester, NY). After complete polymerization (1 hr at 37°C), the matrigel was overlaid with 300 µl standard medium and incubated in a humidified atmosphere of 5% CO^2^ at 37°C for 48 hr. Medium was changed every two days, without disturbing the cell/matrix layer. Phase-contrast images were recorded immediately (0 hr) and at 12-24 hr intervals using a Sony DXC-390 digital camera under an inverted phase microscope (Zeiss Axiovert 200M, Zeiss Inc., Thornwood, NY, USA) to visualize spheroid expansion. Two separate experiments were run in quadruplicate.

The quantification of the area of spheroids has been done as follows: high contrast images of spheroids were captured in NIH ImageJ 1.37v software (Bethesda, MD). A perimeter was established around each spheroid contained in the image. The number of pixels within each perimeter was recorded yielding a pixel number per spheroid, a quantifiable assessment of spheroid "area." Mean pixel number ± SD was measured and exported to Microsoft Excel for further analysis. Invasive outgrowth was quantified by calculating the ratio between the area spheroid after a given incubation period to the original spheroid area at 0h.

**Supplementary references**

Azorsa, D. O., Moog, S., Cazenave, J. P., and Lanza, F. (1999) A general approach to the generation of monoclonal antibodies against members of the tetraspanin superfamily using recombinant GST fusion proteins. *J. Immunol. Methods* **229**, 35– 48.

Bailly M, Bertrand S, Doré JF (1989). Exprimental model of human tumor spontaneous metastasis in immunosuppressed newborn rat.in Immune-deficient Amimals in Experimental Medicine. S Karger, Basel. 140-146.

Bailly M, Doré JF (1992) Clonal drift and role of chromosome dosage in human melanoma metastatic cell lines: a statistical analysis. Anticancer Res. 12(4):1163-72.

Bailly M, Bertrand S, Doré JF.Increased spontaneous mutation rates and prevalence of karyotype abnormalities in highly metastatic human melanoma cell lines.Melanoma Res. 1993.

Jacubovich R, Doré JF (1979). Tumor associated antigens in culture medium of malignant melanoma cell straints. Cancer Immunol. Immunother., **7**, 59-68.

Valéry C, Grob JJ, Verrando P (2001) Identification by cDNA microarray technology of genes modulated by artificial ultraviolet radiation in normal human melanocytes: relation to melanocarcinogenesis. *J Invest Dermatol* **117**, 1471-1482.
